# Coupled Rocking Motion in a Light-Driven Rotary Molecular Motor

**DOI:** 10.1021/acs.joc.2c01830

**Published:** 2022-10-12

**Authors:** Cosima Stähler, Daisy R. S. Pooler, Romain Costil, Dhruv Sudan, Pieter van der Meulen, Ryojun Toyoda, Ben L. Feringa

**Affiliations:** Stratingh Institute for Chemistry, Zernike Institute for Advanced Materials, University of Groningen, Nijenborgh 4, 9747 AG Groningen, The Netherlands

## Abstract

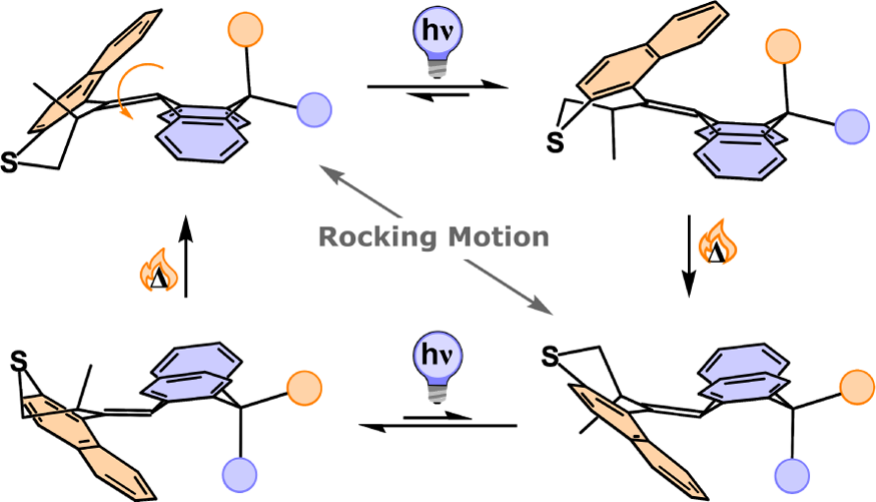

Coupled motion is ubiquitous in Nature as it forms the base for the direction, amplification, propagation, and synchronization of movement. Herein, we present experimental proof for the coupling of the rocking motion of a dihydroanthracene stator moiety with the light-induced rotational movement of an overcrowded alkene-based molecular motor. The motor was desymmetrized, introducing two different alkyl substituents to the stator part of the molecular scaffold, resulting in the formation of two diastereomers with opposite axial chirality. The structure of the two isomers is determined with nuclear Overhauser effect spectroscopy NMR and single-crystal X-ray analysis. The desymmetrization enables the study of the coupled motion, that is, rotation and oscillation, by ^1^H NMR, findings that are further supported by density functional theory calculations. A new handle to regulate the rotational speed of the motor through functionalization in the bottom half was also introduced, as the thermal barrier for thermal helix inversion is found to be largely dependent on the alkyl substituents and its orientation toward the upper half of the motor scaffold. In addition to the commonly observed successive photochemical and thermal steps driving the rotation of the motor, we find that the motor undergoes photochemically driven rotation in three of the four steps of the rotation cycle. Hence, this result extends the scope of molecular motors capable of photon-only rotary behavior.

## Introduction

Molecular machines found in biological systems serve as a great source of inspiration for scientists pursuing molecular nanotechnology. Designed by Nature, biological machines have an innate ability to direct, amplify, and propagate their motion—a feature that is crucial for the emergence of living systems.^1^ Artificial molecular machines developed over the past few decades enable intrinsic motion and allow the transition from simple molecules to dynamic and responsive molecular systems.^2−4^ Light-driven rotary molecular motors are a class of molecules that are able to perform rotational movement upon irradiation with light.^5,6^ The repetitive and unidirectional nature of this rotation allows these motors to operate as nanoscale actuators that may be implemented as key parts in larger molecular machines.^7^ Overcrowded alkene-based molecular motors have already been employed in numerous applications, ranging from biological systems to smart materials.^8−12^

Typically, the rotation cycle of a molecular motor entails the sequential formation of four different isomers—two thermally stable isomers and two metastable isomers, which over time convert into the thermally stable isomers.^6,13^ When irradiated with light, the central double bond, that is, the axle of rotation, undergoes a photochemical *E*-*Z* (*PEZ*) isomerization. In this step, the stereogenic methyl group in the upper half adopts a *pseudo*-equatorial position, which is less favored than the thermally stable *pseudo*-axial position—hence the term metastable. When relaxing to the following stable state, the molecule undergoes a thermal helix inversion (THI). Repetition of the *PEZ* and THI processes instigates a sequential population of the four isomers, completing a fully unidirectional 360° rotation cycle about the central double bond axis, and continuous irradiation allows repetitive rotary motion. Exploring coupled motion is a worthwhile research venture to introduce complexity in tasks performed by artificial (supra-)molecular machines, similar to the function of their biological counterparts.^14^ Recently in our group, we have observed computationally that the rotation of the motor can be coupled to the paddling movement of a dibenzofluorenyl moiety^15^ and shown that the rotation of a biphenyl rotor can be interlocked and synchronized with the rotation of the motor.^16^ Recently, Dube and co-workers have shown that the rotation of molecular motors based on hemithioindigo (HTI) can control the rotation around a remote biaryl axis.^17,18^ Here, we present experimental evidence that the conformational change of a 9,10-dihydroanthracene (DHA) stator moiety in a molecular motor is coupled with and controlled by the rotational motion of the rotor moiety. In contrast to coupling the motor rotation to a secondary rotary motion^16^ or to translational motion,^19^ synchronization of a rotary motor with an oscillating/rocking motion remains to be demonstrated experimentally.^20^

**Figure 1:**
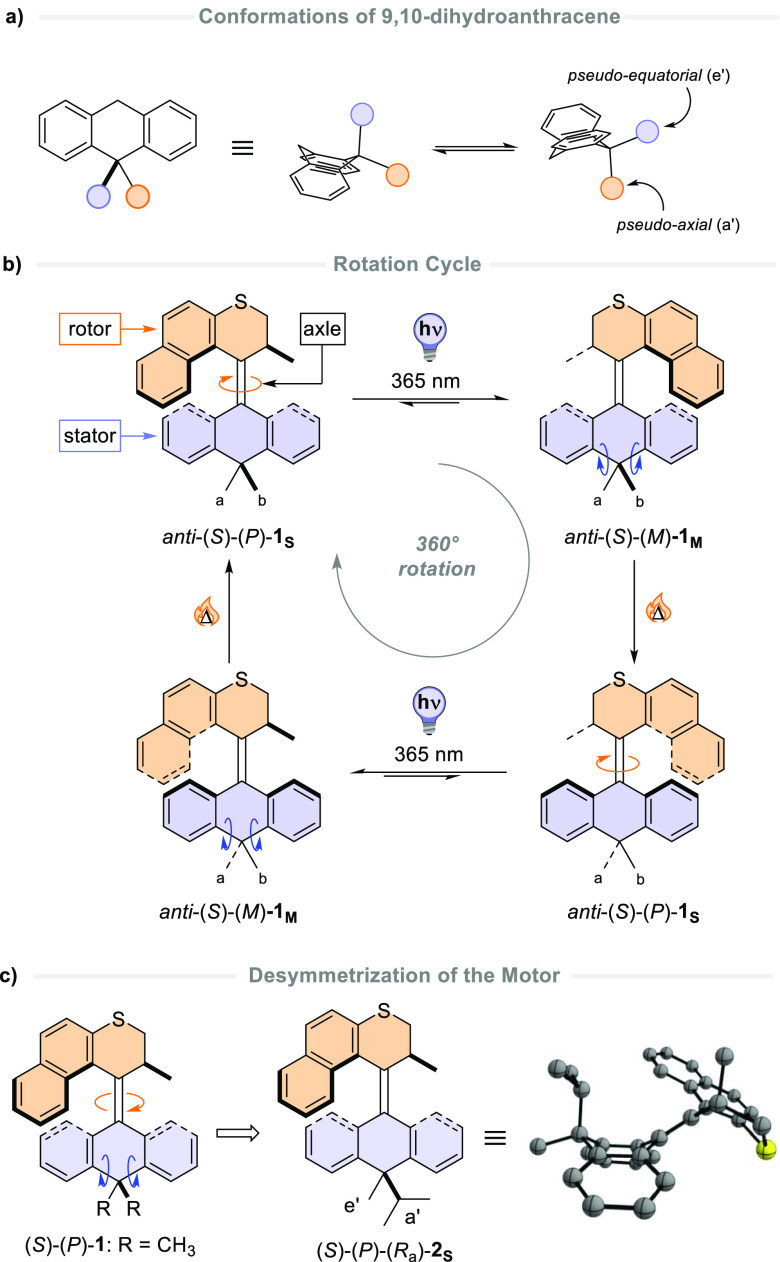
(a) Conformations of DHA; (b) rotational cycle of motor **1**; and (c) desymmetrization of motor **1** to afford motor **2**, with the DFT-simulated structure of (*S*)-(*P*)-(*R*_a_)-**2**_**S**_ [B3LYP/6-31G(d,p)].

Benzannulated analogues of cyclohexane, like DHA, display a restricted number of conformations. DHA is non-planar and exists as rapidly interconverting boat or “butterfly-shaped” conformers (Figure 1a).^21^ Studies of the stereochemical properties of DHA have revealed their deviations from the typical conformations of cyclohexane.^22^ Interestingly, the steric hindrance provided by the flanking aryl rings destabilizes the conformer in which the larger substituent at the sp^3^ carbon is in a *pseudo*-equatorial position, strongly favoring the *pseudo*-axial conformer instead.^23^ The introduction of an exocyclic double bond at the 9-position of DHA can significantly increase the barrier of interconversion between the *pseudo*-axial and *pseudo*-equatorial isomer of the sp^3^ carbon, giving rise to conformers with enhanced kinetic stability.^24,25^

We previously reported molecular motor **1** with a lower half based on DHA (Figure 1b),^20,26^ and it was proven that this motor retained its rotary motion while attached to a gold surface.^27^ Although the butterfly conformation of the lower half can theoretically produce two conformers (namely *anti*- and *syn*-folded), only one species with the flanking rings in the stator pointing away from the top half rotor part (*anti*-folded) was observed.^26^ While studying the conformational interconversion during the rotation of this molecular motor computationally, it was found that the DHA moiety undergoes a conformational change at its sp^3^-hybridized carbon atom at each half-turn to invert the configuration of the ring,^20^ placing a distinct methyl group (Me_a_ when *anti*-(*S*)-(*P*)-**1**_**s**_ is formed and Me_b_ when *anti*-(*S*)-(*P*)-**1**_**s**_ is formed) in the *pseudo*-axial position. Consequently, motor **1** has the propensity to perform a coupled motion of its bottom half during its unidirectional rotation, which is herein referred to as rocking motion (Figure 1b).^20^

Given the current challenge to design systems that enable coupled motion in advanced molecular machines to ultimately produce more complex mechanical functions at the nanoscale,^14^ we aimed to establish the coupled rocking motion directed through remote conformational control. In our new design, we desymmetrized the bottom half of compound **1** by substituting two different alkyl groups, an *iso*-propyl group and a methyl group, at the sp^3^-hybridized carbon atom (**2**, Figure 1c). This introduces an element of axial chirality^28^ in the molecular motor which is independent from the helicity of the molecule, denoted by *R*_a_/*S*_a_.^29^ By correlating the ^1^H NMR chemical shift of the alkyl substituents to their relative conformation in DHA analogues,^25,30,31^ together with density functional theory (DFT) calculations and X-ray crystal structural analysis, we demonstrate that the unidirectional rotation of molecular motor **2** is coupled to a rocking motion of its lower half through controlled folding of the DHA moiety (Figure 1a).

## Results and Discussion

To prove the change in relative conformation of the two substituents at the quaternary carbon, target motor **2** was designed. The bottom half is desymmetrized by replacing one of the methyl groups in **1** with an *iso-*propyl group (Figure 1c). The difference in steric demand between the two alkyl groups raises the possibility to separate axial diastereomers using standard purification techniques, while the diastereotopic *iso-*propyl group provides a handle to investigate the stereodynamic properties of the folding DHA stator by NMR spectroscopy.

**Figure 2:**
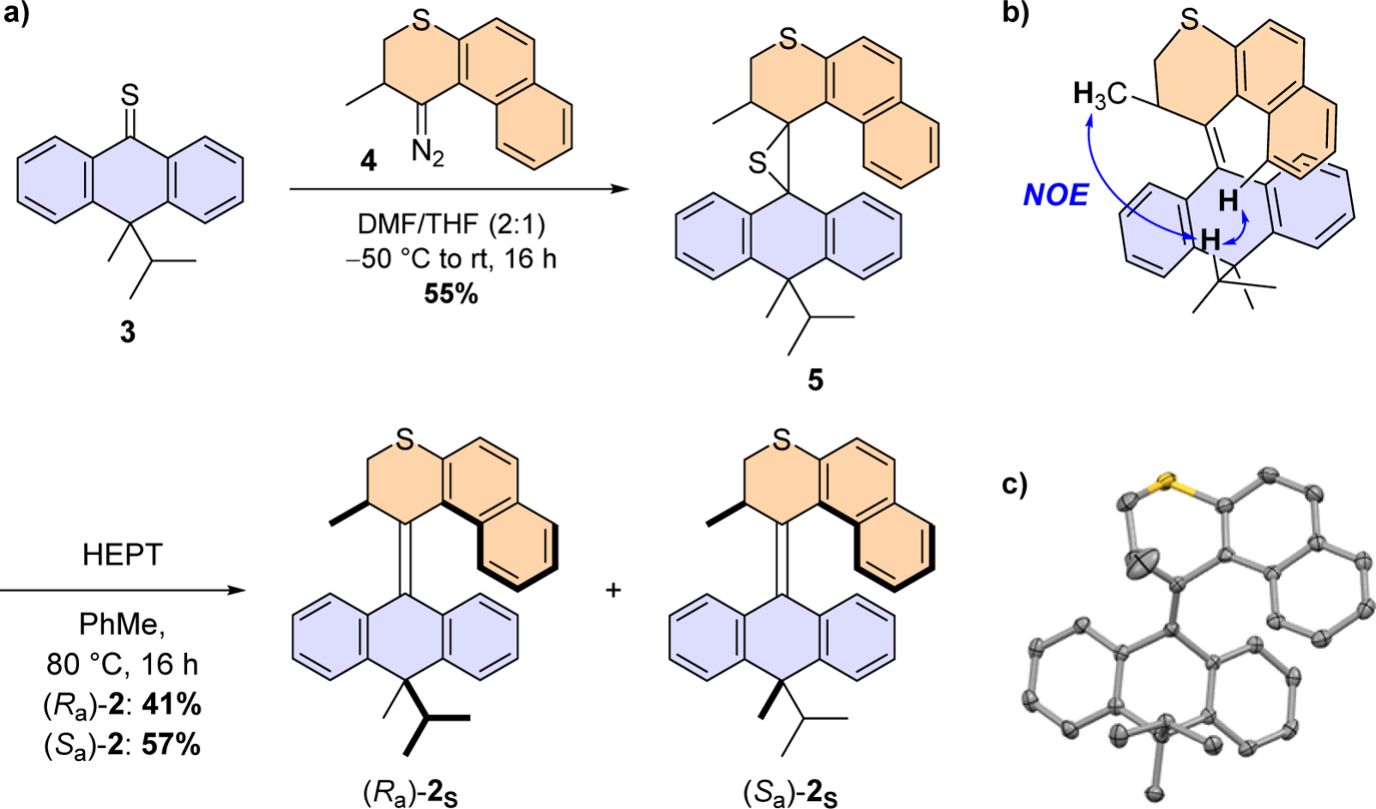
(a) Synthetic procedure for motor **2**; (b) relative configuration assignment of (*R*_a_)-**2**_**S**_ by ^1^H NOESY NMR; and (c) ORTEP image (ellipsoid probability at 50%) of the X-ray crystal structure of (*R*_a_)-**2**_**S**_. Protons are omitted for clarity.

Molecular motor **2** was prepared by a Barton–Kellogg coupling of thioketone **3** (prepared from DHA using procedures developed by Rabideau and co-workers^32^) and diazo compound **4** in good yield, in a nearly equal mixture of racemic diastereomers (Figure 2a). The reaction initially leads to the formation of episulfide **5**, which is transformed to motor **2** through sulfur removal by addition of hexaethyl phosphorous triamide (HEPT). The *S*_a_-**2**_**S**_ and *R*_a_-**2**_**S**_ diastereomers could be separated by flash column chromatography.

The relative configuration of both diastereomers was assigned using ^1^H nuclear Overhauser effect spectroscopy (NOESY) NMR analysis. Through-space coupling was observed between the stereogenic methyl group in the upper half and the *iso*-propyl group in the lower half of (*R*_a_)-**2**_**S**_ and with the aromatic protons of the naphthalene upper half (Figure 2b), confirming the *syn* arrangement of the upper half with the bulky *iso*-propyl group. This is further supported by the upfield shift of the ^13^C NMR signal of the lower-half methyl group, indicative of the equatorial orientation of the substituent (see Supporting Information, NMR spectra).^33^ The alkyl substituents of the lower half of (*S*_a_)-**2**_**S**_ show through-space coupling to the aromatic protons of the lower half, but no coupling to the upper half was observed (see Supporting Information, NMR spectra). Interestingly, the lower-half methyl group is also particularly deshielded in the ^13^C NMR spectrum of (*S*_a_)-**2**_**S**_, suggesting that the structure of this diastereomer deviates from that of (*R*_a_)-**2**_**S**_.

The relative stereochemistry of (*R*_a_)-**2**_**S**_ was confirmed by its solid-state structure. Crystals suitable for X-ray diffraction were grown by slow diffusion of pentane into a concentrated solution of (*R*_a_)-**2**_**S**_ in dichloromethane (Figure 2c). In the solid state, (*R*_a_)-**2**_**S**_ adopts an *anti-*folded state typical of structurally related molecular motors.^29^ The steric hindrance brought about by the upper half forces the lower half to adopt a butterfly conformation, placing the *iso-*propyl substituent in a favored *pseudo*-axial position; the folding angle between the two phenyl rings in the lower half is 128°, in good agreement with the DFT-calculated angles for motors **1**(20) and **2**, being 132 and 127°, respectively (see Supporting Information, Figure S1).

The first evidence for the potential rocking motion was demonstrated by analysis of the ^1^H NMR spectrum of motor **1**. At −35 °C in tetrachloroethane-*d*_2_, the lower-half methyl groups are well separated into two singlets at 1.78 and 1.88 ppm (see Supporting Information, Figure S3). This correlates well to one of the Me substituents adopting a *pseudo*-axial (downfield shift) and the other one a *pseudo*-equatorial (upfield shift) orientation in the stable isomer of the motor.^30^ Upon irradiation, a metastable isomer whose methyl signals are much less separated is formed (Δδ = 0.03 ppm). This close magnetic equivalence is possibly due to a near-planar conformation of the bottom half in the metastable isomer.

**Figure 3:**
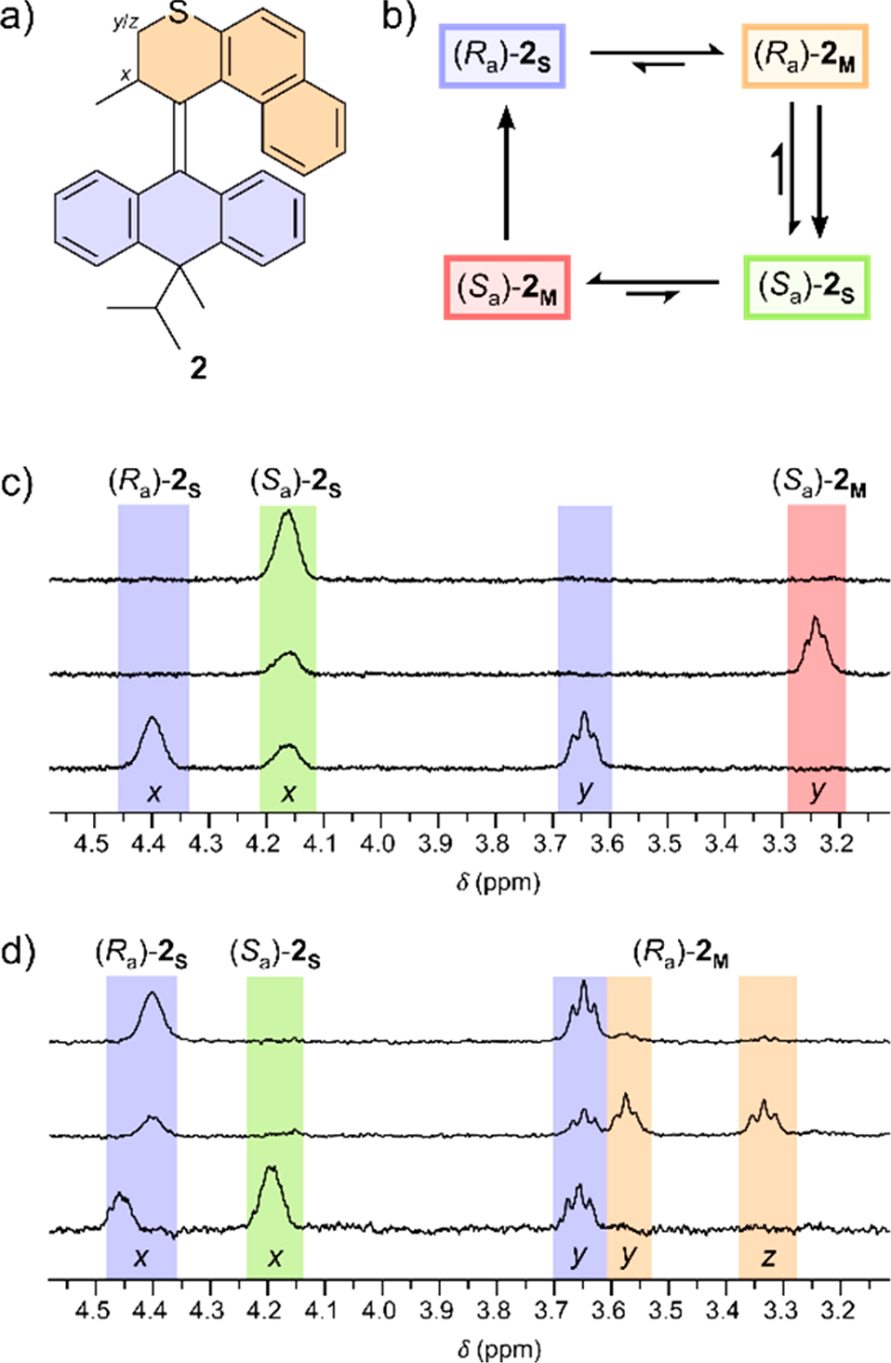
(a) Structure of **2** with indication of hydrogen atoms followed in ^1^H NMR; (b) schematic representation of the rotational cycle with indication of the color coding used for the ^1^H NMR spectra; and (c) selected region of the ^1^H NMR spectrum of (*S*_a_)-**2**_**S**_ in tetrachloroethane-*d*_2_ at −35 °C (top). (*S*_a_)-**2**_**M**_ forms upon irradiation at 365 nm, and a PSS of 68:32 ((*S*_a_)-**2**_**M**_: (*S*_a_)-**2**_**S**_) establishes after 1 h of irradiation (middle) and after THI after keeping in the dark at room temperature for 24 h and formation of (*R*_a_)-**2**_**S**_ (bottom). (d) Selected region of the ^1^H NMR spectrum of (*R*_a_)-**2**_**S**_ in tetrachloroethane-*d*_2_ at −35 °C (top), after irradiation at 365 nm for 30 min inducing formation of (*R*_a_)-**2**_**M**_ (middle), and after THI during keeping in the dark at 75 °C overnight, forming (*S*_a_)-**2**_**S**_ (bottom). Hydrogen atoms are assigned in Figure 3a.

The capability of **2** to perform unidirectional rotary motion under light irradiation (Figure 3) was investigated by ^1^H NMR. Upon in situ irradiation of (*S*_a_)-**2**_**S**_ at 0 °C in tetrachloroethane-*d*_2_ at 365 nm, a new set of signals appeared. The most significant shifts were observed for the protons in the allylic position (H_*x*_) and the methylene group (H_*y*_ and H_*z*_), see Figure 3a for hydrogen atom labeling. The formation of the new signals is indicative of the formation of (*S*_a_)-**2**_**M**_, appearing with a photostationary state (PSS) of 68:32 [(*S*_a_)-**2**_**M**_: (*S*_a_)-**2**_**S**_] after 1 h of irradiation (Figure 3c). When keeping the sample in the dark at room temperature for 24 h, this newly formed metastable isomer undergoes selective THI with concomitant formation of (*R*_a_)-**2**_**S**_, proving that the first half of the rotational cycle is unidirectional. Eyring analysis in toluene-*d*_8_ provides a barrier to THI of Δ*G*^‡^_293_*_K_* = 89.3 kJ mol^–1^ correlating to a half-life of 0.26 h at 20 °C (for details, refer to Supporting Information, Figure S10 and Table S2). This value is significantly lower than that for parent motor **1**, which has a half-life of about 9 days at 20 °C (Δ*G*^‡^_293 K_ = 106 kJ mol^–1^).^26^

In situ irradiation of (*R*_a_)-**2**_**S**_ at 365 nm at −35 °C in tetrachloroethane-*d*_2_^1^H NMR (Figure 3d) revealed the formation of (*R*_a_)-**2**_**M**_. The sample was kept at 75 °C overnight to induce THI, showing the formation of (*S*_a_)-**2**_**S**_, thus proving the unidirectionality of the second half of the 360° rotation cycle. The energy barrier for this THI was considerably higher than that for (*S*_a_)-**2**_**M**_. Eyring analysis in toluene-*d*_8_ reveals a barrier to THI of Δ*G*^‡^_293 K_ = 108.5 kJ mol^–1^ correlating to a half-life of 28 days at 20 °C (for details, see Supporting Information, Figure S11 and Table S3), also correlating to the THI barrier for parent motor **1** (ΔG^‡^_293 K_ = 106 kJ mol^–1^).^26^ The substantially longer half-life of compound (*R*_a_)-**2**_**M**_ compared to that of (*S*_a_)-**2**_**M**_ is attributed to the orientation of the *iso*-propyl substituent. During the THI of (*R*_a_)-**2**_**M**_, the bulkier *iso*-propyl group has to adopt the less preferred *pseudo*-equatorial position. Conversely, during the THI of (*S*_a_)-**2**_**M**_, the *iso*-propyl group adapts from the *pseudo*-equatorial orientation to its favored *pseudo*-axial orientation, speeding up the thermal process. This difference allows the speed of both 180° turns of motor **2** to be individually tuned, a result that is not typically seen in second-generation molecular motors.

**Figure 4:**
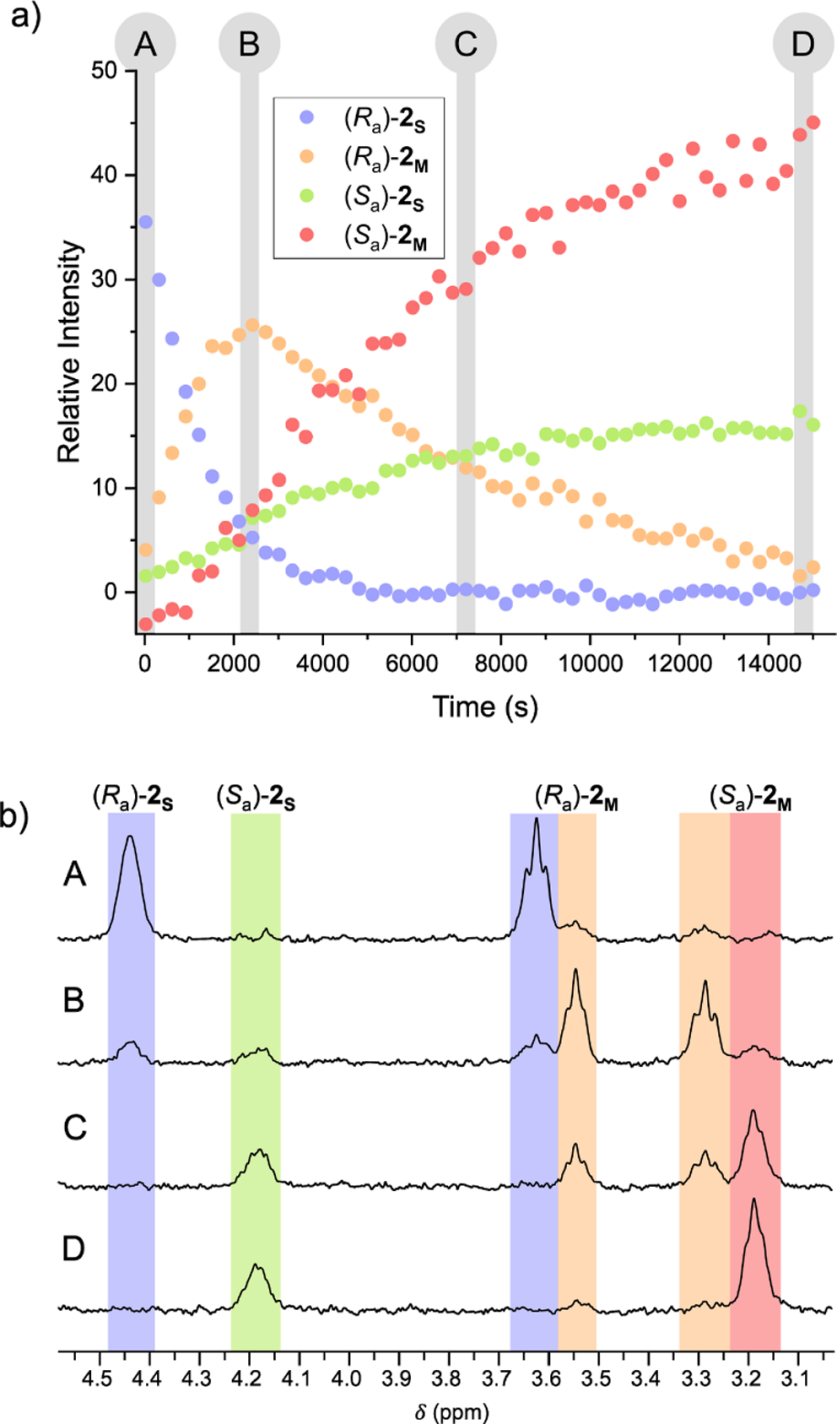
(a) Photokinetic profile of the irradiation of (*R*_a_)-**2**_**S**_ at 365 nm in tetrachloroethane-*d*_2_ at −35 °C observed by ^1^H NMR spectroscopy. Intensities are given relative to the normalized integration of the NMR signal. (b) Representative selected regions of ^1^H- NMR spectra recorded during the prolonged irradiation. Time stamps are indicated in the photokinetic profile on top.

Perhaps more interestingly, upon prolonged irradiation of metastable isomer (*R*_a_)-**2**_**M**_, new peaks are observed in the ^1^H NMR spectrum to yield the same product distribution as that yielded when irradiating (*S*_a_)-**2**_**S**_ (Figure 4a). Photokinetic analysis revealed that (*S*_a_)-**2**_**S**_ was generated through a photochemical helix inversion of (*R*_a_)-**2**_**M**_, a behavior which was previously observed in related molecular motors.^29,34^ Starting the irradiation at 365 nm in tetrachloroethane-*d*_2_ at −35 °C from pure (*R*_a_)-**2**_**S**_ (Figure 4b, A), the metastable isomer (*R*_a_)-**2**_**M**_ forms and reaches its maximum molar ratio after ∼30 min of irradiation (Figure 4b, B). Subsequently, (*R*_a_)-**2**_**M**_ decays and disappears completely after ∼2 h of irradiation (Figure 4b, C, D). During that time, the formation of (*S*_a_)-**2**_**S**_ can be observed and is followed by the formation of (*S*_a_)-**2**_**M**_ after a short lag time. After 2 h, the same photostationary distribution (68:32) is established as that for the irradiation experiment starting from (*S*_a_)-**2**_**S**_ (Figure 4b, D).

**Figure 5:**
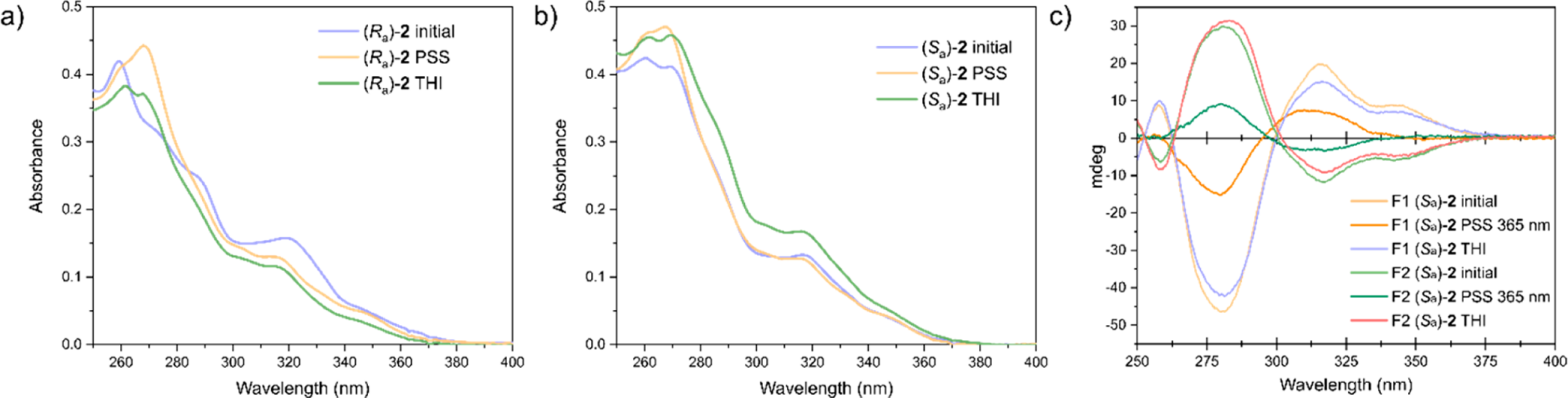
(a) UV–vis spectra of (*R*_*a*_)-**2** in dichloroethane (10^–5^m) initially (purple), after irradiation at 365 nm for 12 min at 10 °C (orange), and after keeping in the dark for 50 min at 10 °C (green); (b) UV–vis spectra of (*S*_*a*_)-**2** in dichloroethane (10^–5^m) initially (purple), after irradiation at 365 nm for 2 min at 10 °C (orange), and after keeping in the dark for 25 min at 20 °C (green); and (c) CD spectra of the first eluted fraction (F1) of (*S*_a_)-**2**_**S**_ and the second eluted fraction (F2) of (*S*_*a*_)-**2**_**S**_ in dichloroethane at room temperature, after irradiation at 365 nm, and after THI after keeping in the dark at room temperature.

The dynamic properties of (*S*_a_)-**2**_**S**_ and (*R*_a_)-**2**_**S**_ were also investigated by UV–vis spectroscopy (Figure 5a,b). Both isomers show absorption maxima at around 270 and 320 nm, in line with previously reported UV–vis spectra of related motor structures.^26^ Upon irradiation at 365 nm, a minor shift of the absorption maxima was observed, which was reversed upon keeping the samples in the dark at 20 °C, indicating that a reversible photoinduced process is taking place. (*S*)-(*P*)-(*S*_a_)-**2**_**S**_ and (*R*)-(*M*)-(*S*_a_)-**2**_**S**_ enantiomers (two eluted fractions, F1 and F2) could further be separated by chiral supercritical fluid chromatography (SFC) and studied by circular dichroism (CD) spectroscopy in dichloroethane (Figure 5c). Both enantiomers initially show a Cotton effect of opposite signs, exhibiting their opposite helicity. Upon irradiation at 365 nm, the intensity of the signals decreases, indicative of the formation of the metastable isomers of opposite helicity in the mixture with the stable state at the photostationary state. The intensity increased again upon keeping the samples at room temperature, showing that the motors thermally relax to adopt their initial helicity.

**Figure 6:**
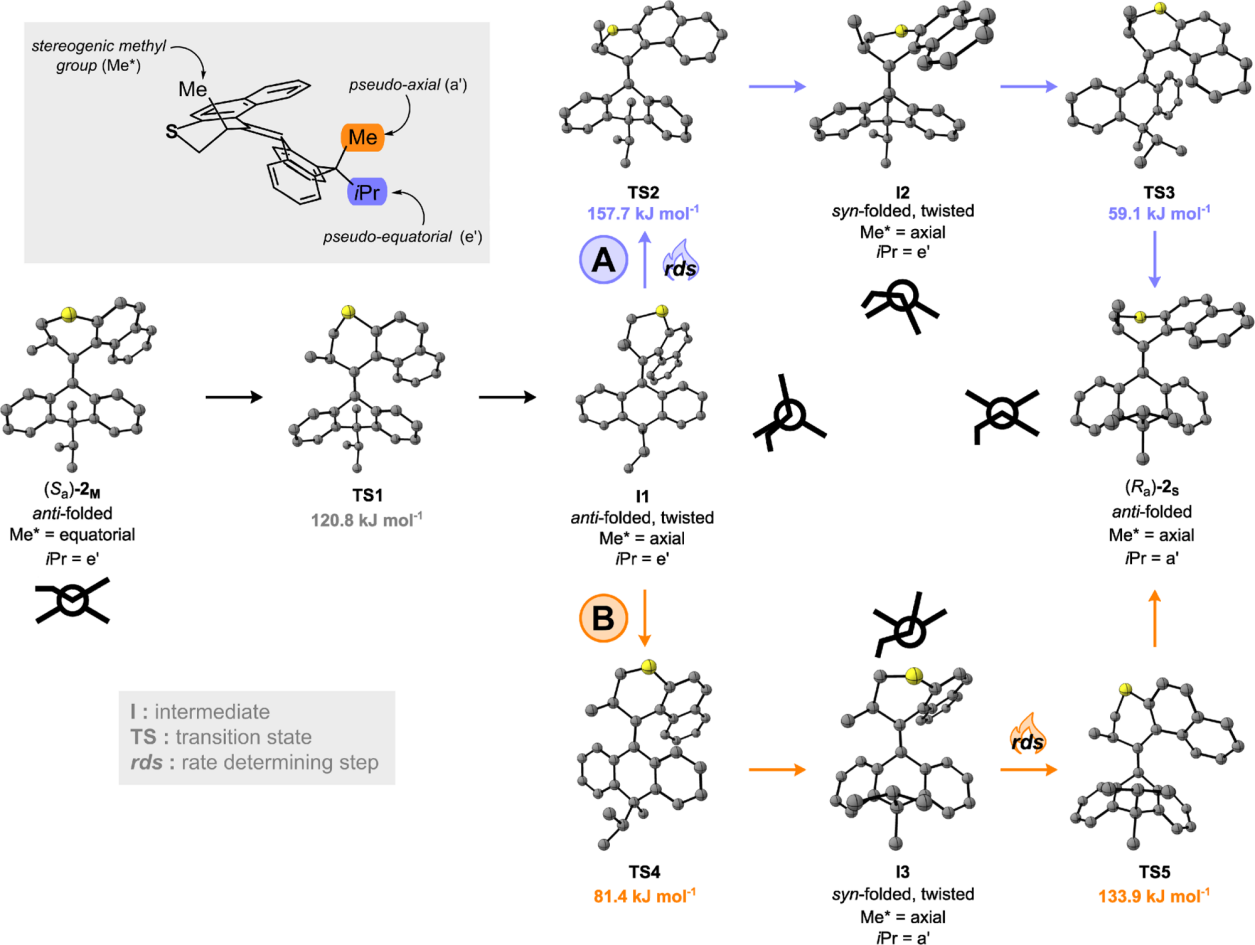
Thermal conversion from metastable (*S*_a_)-**2**_**M**_ to stable (*R*_a_)-**2**_S_ via pathways A (top, purple) and B (bottom, orange). For all intermediates, a schematic top view of the molecule along the central double bond axis of the motor is shown. For all transition states, the energy barriers are quoted in kJ mol^–1^. The rate-determining ring flip in the upper half is indicated with *rds*.

Having demonstrated the rotational cycle of motor **2** experimentally, we investigated the conformational changes in the lower half through DFT calculations. The geometries of the ground state minima of motor **2** were observed at the B3LYP/6-31G(d,p) level of theory, which has previously afforded reliable energies and geometries for structurally related overcrowded alkene-based molecular motors.^20^ As determined by X-ray analysis (vide supra), motor (*R*_a_)-**2**_**S**_ exists in an *anti*-folded butterfly conformation with the stereogenic allylic methyl group on the upper half in the *pseudo*-axial position, similar to structurally related molecular motors (Figure 6).^20,26^ In the ground state, the *iso*-propyl group of the DHA moiety is oriented *syn* to the upper half, adopting a favored *pseudo*-axial conformation. A root mean square deviation was performed to compare these calculations with the X-ray structure of (*R*_a_)-**2**_**S**_, and the value was 0.39 Å (see Supporting Information, Figure S2). As has been observed with molecular motors with six-membered rings in their upper halves, the majority of the deviation between the experimental and simulated structures is centered at the methylene group which can induce puckering, resulting in multiple possible conformers.^35^

Interestingly, (*S*_a_)-**2**_**S**_ can exist in the *syn*-folded conformation (**I2**, in Figures S13 and S14), which is typically disfavored in second-generation molecular motors.^20^ Presumably, the preference of the *iso*-propyl group for a *pseudo*-axial conformation competes with the usually favored *anti*-folded conformation of the stable isomer of molecular motors. The contribution of this bulky *iso*-propyl group is considerable, as the *syn*-folded conformation is 19.19 kJ mol^–1^ more stable, which preserves the relative stereochemistry of the lower half to that of (*R*_a_)-**2**_**S**_. This supports the absence of NOE correlations in the NMR spectrum of (*S*_a_)-**2**_**S**_ (see Supporting Information, Figures S16 and S17) and the similar upfield shift of the lower-half methyl group in the ^13^C NMR spectra of both (*S*_a_)-**2**_**S**_ and (*R*_a_)-**2**_**S**_.

Both metastable states (*R*_a_)-**2**_**M**_ and (*S*_a_)-**2**_**M**_ exist in *anti*-folded butterfly conformations with the stereogenic methyl group on the upper rotor half in the *pseudo*-equatorial position. Seemingly, the presence of steric bulk in the lower stator half does not prevent (*S*_a_)-**2**_**M**_ from adopting a conformation in which the *iso-*propyl moiety lies in a *pseudo*-equatorial position. This information is crucial for the remote control of motion, as this means that the relative configuration of the axially chiral moiety is inverted during the whole rotational cycle. The (*S*_a_)-**2**_**M**_ isomer is particularly destabilized due to a combination of both the *iso*-propyl group and the stereogenic methyl group of the upper rotor half being in *pseudo*-equatorial positions (see Supporting Information, Table S5). Therefore, the experimental energy barrier for the THI is lower (Δ*G*^‡^_293 K_ = 89.3 kJ mol^–1^) as this metastable isomer is more thermally labile. These findings are reflected in the thermal barriers calculated for this motor (see Supporting Information, Table S6).

Similarly to motor **1**, the thermal relaxation steps from (*S*_a_)-**2**_**M**_ to (*R*_a_)-**2**_**S**_ occur via a multi-step process (Figure 6).^20^ Starting from (*S*_a_)-**2**_**M**_, there is initially a ring flip of the thiane upper half (via **TS1**) which brings the stereogenic methyl group to the *pseudo*-axial position, reaching the *anti*-folded, twisted intermediate **I1**. Next, there are two pathways possible: either (A) the upper half slips over the lower half via **TS2**, giving *syn*-folded twisted **I2**, followed by a ring flip in the lower half (via **TS3**) to give the stable (*R*_a_)-**2**_**S**_ state or (B) the ring flip in the lower half occurs first, affording *syn*-folded twisted **I3** via **TS4**, and then, subsequently, the upper half slips over the lower half (via **TS5**) to generate (*R*_a_)-**2**_**S**_. In this way, during the THI steps, a ring flip of the lower half is paired with the upper half slipping over the lower half. This inversion of configuration, repeatedly occurring at each revolution, couples the rotation of the molecular motor with a remote, “rocking” motion of the DHA lower half.^20^

**Figure 7:**
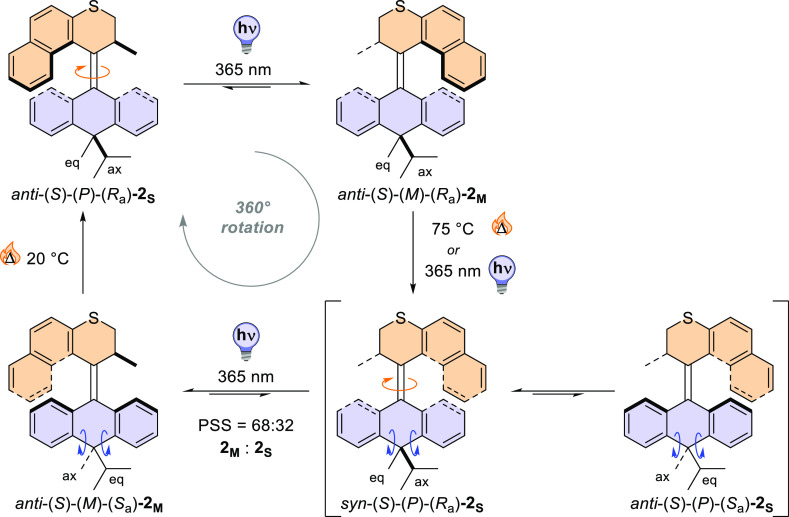
Proposed mechanism for the rotational cycle of **2** with a coupled rocking motion.

The proposed rotational mechanism is summarized in Figure 7. Based on the presented experimental data and computational study, it is shown that the rotation of the upper half and the axial/equatorial interconversion of the *iso-*propyl and methyl substituents in the lower half are synchronized, demonstrating a coupled rocking motion.

## Conclusions

We have presented a new molecular motor with distinct alkyl substituents in the bottom half. The substitution pattern results in the occurrence of two diastereomeric structures with opposite axial chirality. The diastereoisomers can be sequentially interconverted through photochemical and thermal steps, as is typically the case for overcrowded alkene-based molecular motors. In addition, we found that conversion between isomers can occur via a progression of photochemical steps, as previously reported for a limited number of cases.^29,34^ In this work, we have shown through thermal and photochemical studies by NMR spectroscopy and DFT calculations that the rocking motion^20^ of the bottom stator half is coupled to the rotational movement of the upper rotor half of the molecular motor. Formally, control of the unidirectional rotation of a C=C double bond can be used to remotely control the axial chirality of a DHA moiety. Continuous processing of the motor thus generates a synchronized rocking motion.

Furthermore, we observe that the steric hindrance of the alkyl substituents and their position with respect to the flanking phenyl rings and the upper half of the motor has a substantial influence on the half-life of the thermal steps of the rotation. This can serve as a new tool for regulation of the thermal barrier for the helix inversion of molecular motors, which could enable the access to a wider variety of motors with different rotation speeds.

## Supporting Information Available

Synthetic procedures, experimental conditions for UV–vis and CD studies, in situ irradiation NMR studies, computational details, and ^1^H and ^13^C NMR spectra (PDF)
All computed structures provided as .xyz files (ZIP)
