# Supplementary material for: Coupled Rocking Motion in a Light-Driven Rotary Molecular Motor
Source: J Org Chem. 2022 Oct 12;89(1):1–8. doi: 10.1021/acs.joc.2c01830 (PMC10777401; doi:10.1021/acs.joc.2c01830)
Supplement: Supplementary file 1 — jo2c01830_si_001.pdf [file jo2c01830_si_001.pdf]

Supporting Information: Coupled Rocking Motion in a Light-Driven Rotary Molecular Motor

Cosima Stähler, Daisy R. S. Pooler, Romain Costil,* Dhruv Sudan, Pieter van der Meulen, Ryojun Toyoda and Ben L. Feringa*

Stratingh Institute for Chemistry, University of Groningen, 9747 AG Groningen, The Netherlands

Email: r.p.s.costil@rug.nl, b.l.feringa@rug.nl

Table of contents

General Methods S2

Synthesis S3

X-ray crystal structure of (R_a)-2_S S12

Photochemical and Thermal Isomerisation Studies S15

Eyring Analysis S19

Computational analysis S23

Conformational Analysis by NOESY S27

References: S30

NMR spectra S31

General Methods

All reagents were purchased from commercial sources and used as received without further purification. Dry solvents were obtained from a MBraun SPS-800 solvent purification system. The solvents used for spectroscopic studies were of spectroscopic grade (UVASOL, Merck).

Heated reactions were performed in an oil bath regulated at the appropriate temperature. Cooling to -78 °C was achieved in an acetone bath with dry ice.

Column chromatography was performed on silica gel (Merck, type 9385, 230-400 mesh) or on a Büchi Reveleris X2 Flash Chromatography system using Büchi silica cartridges. Thin layer chromatography (TLC) was carried out on aluminium sheets coated with silica gel (60 F254, 0.25 mm, Merck). Compounds were visualized with a UV lamp (254 nm) and/or by staining with a Cerium Ammonium Molybdate solution.

High Resolution Mass spectra (HRMS) were recorded on an LTQ Orbitrap XL or an Orbitrap Velos Pro (Thermo Scientific) mass spectrometer connected to an LC20 HPLC system (Shimadzu).

NMR spectra were obtained using a Varian Oxford NMR (^{1}H: 300 MHz) with FTS Systems Air Jet temperature control, a Varian Mercury Plus (^{1}H: 400 MHz, ^{13}C: 100 MHz), a Varian Unity Plus (^{1}H: 500 MHz, ^{13}C: 125 MHz) with FTS Systems Air Jet temperature control or a Bruker Avance (^{1}H: 600 MHz, ^{13}C: 151 MHz). Chemical shifts of ^{1}H and ^{13}C nuclei are reported in δ units (ppm) relative to the residual solvent signal (^{1}H: $CDCl_3$ = 7.26 ppm, C_6D_6 = 7.16 ppm, $C_2D_2Cl_4$ = 6.00 ppm, toluene-d8 = 2.09 ppm; ^{13}C $CDCl_3$ = 77.0 ppm, C_6D_6 = 128.0 ppm). The splitting patterns are designated as follows: s (singlet), d (doublet), t (triplet), q (quartet), dd (doublet of doublets), dt (doublet of triplets), ddd (doublet of doublets of doublets), dtd (doublet of triplets of doublets), ddt (doublet of doublets of triplets), td (triplet of doublets), and m (multiplet).

Supercritical Fluid Chromatography (SFC) was performed on a TharTechnologies, Inc. (Waters) Investigator II system.

UV/Vis absorption spectra were recorded on an Agilent 8453 UV/Vis Diode Array System, equipped with a Quantum Northwest Peltier controller, in 10 mm quartz cuvettes.

CD spectra were measured on a Jasco J-815 CD spectrometer.

Irradiations were performed using LED lamps obtained from ThorLabs (M365F1), either directly aimed at a reaction solution or coupled to a fiber optic cable inserted into an NMR tube for *in-situ* NMR irradiation experiments.

Synthesis

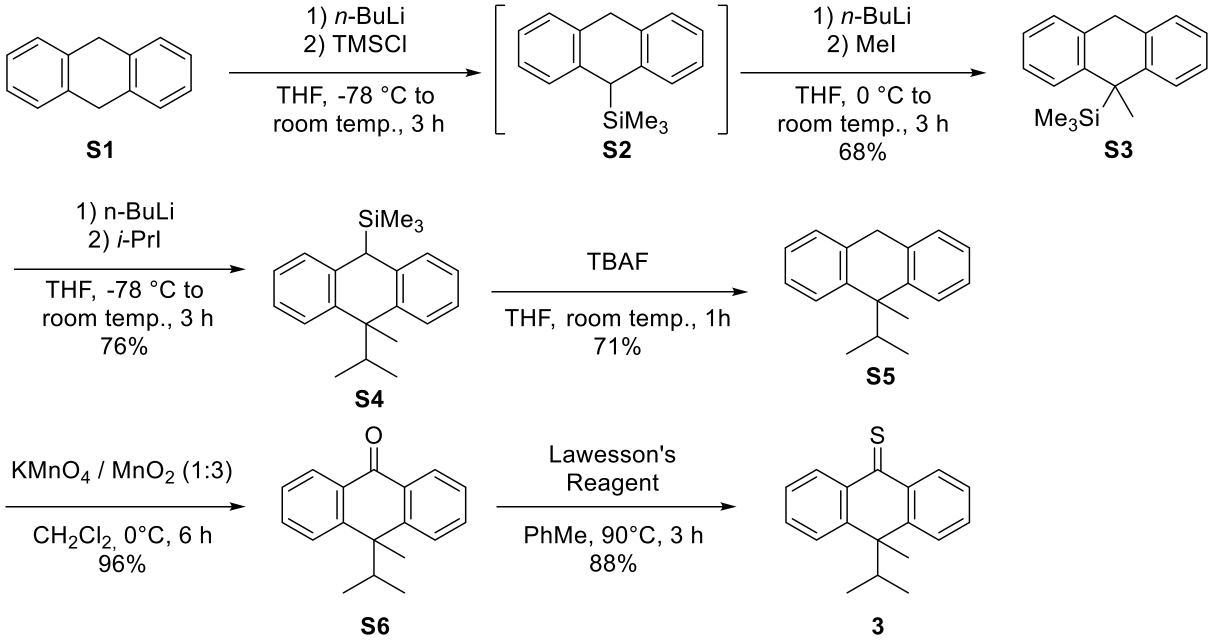

*Scheme S 1: Synthesis scheme for the lower half encompasses a series of alkylations, an oxidation and the formation of a thioketone **3**.* [6,7]

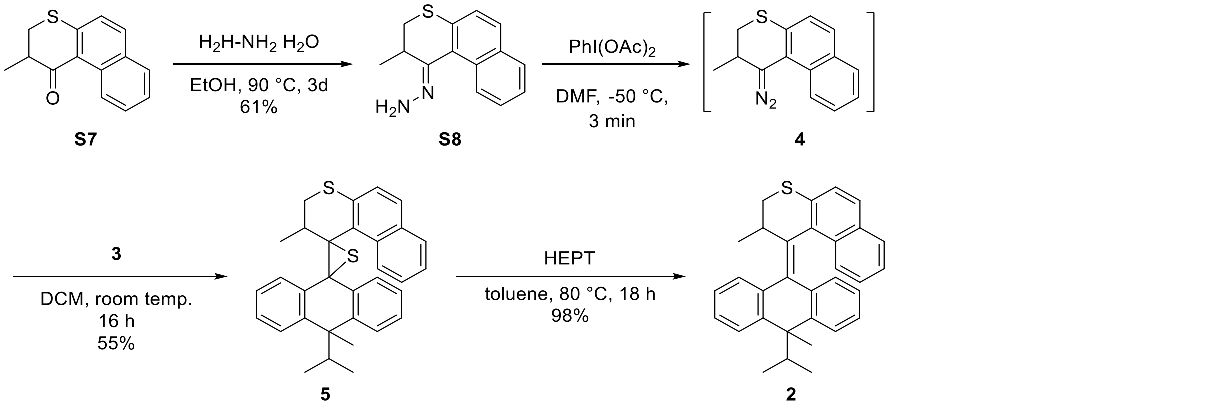

*Scheme S 2: Synthesis Scheme for the Barton-Kellogg reaction which will give the final product **2**.*

1-(10,10-Dimethylanthracen-9(10*H*)-ylidene)-2-methyl-2,3-dihydro-1*H*-benzo[*f*]thio-chromene (1)

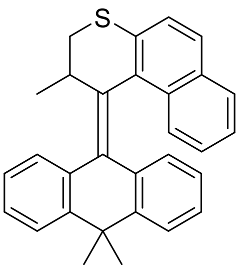

1

Motor 1 was synthesised according to literature procedures.[1]

10-Methyl-10-(trimethylsilyl)-9,10-dihydroanthracene (S3)

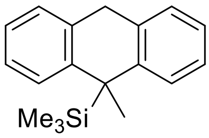

S3

9,10-Dihydroanthracene (**S1,** 8.00 g, 10.0 mmol) was dissolved in THF (40.0 mL, 0.25 M), under an inert atmosphere and the solution was cooled down to –78 °C. *n*-Butyllithium (7.40 mL, 1.63 M in hexane, 12.0 mmol) was added dropwise and the reaction mixture was stirred for 1 h at – 70 °C. A solution of TMSCl (1.5 mL, 12.0 mmol) in THF (10.0 mL, 1.2 M) was added to the reaction mixture, which was allowed to warm to room temperature and then was stirred for 2 h. The mixture was cooled down to 0 °C and *n*-butyllithium (7.4 mL, 1.63 M in hexane, 12.0 mmol) was added dropwise and the solution was stirred for 1 h at 0 °C. Subsequently, methyl iodide (0.74 mL, 12.0 mmol) was added and the reaction mixture was stirred at room temperature for 3 h. Upon completion, diethyl ether (100 mL) and water (100 mL) were added and the phases were separated. The organic phase was washed twice with water (100 mL), once with brine (100 mL) and then dried over $MgSO_4$ and filtered over a silica pad. The solvent was removed under reduced pressure. The residue was recrystallised from methanol and was the product was obtained as white crystals (7.99 g, 29.9 mmol, 68% yield). The data is in accordance with the literature.[2]

^{1}H NMR (400 MHz, $CDCl_3$) δ 7.35 (d, *J* = 7.9 Hz, 2H), 7.23 (m, 4H), 7.14 (t, *J* = 7.3 Hz, 2H), 4.14 (d, *J* = 19.4 Hz 1H), 3.96 (d, *J* = 19.4 Hz, 1H), 1.83 (s, 3H), -0.14 (s, 9H).

^{13}C{^{1}H} NMR (101 MHz, $CDCl_3$) δ 141.6, 134.1, 128.0, 126.1, 125.9, 125.0, 36.8, 36.2, 20.9, -2.8.

HRMS (APCI pos) calcd. $C_{18}H_{23}Si$ $[M+H]^+$ 267.1569, found 267.1564.

10-Methyl-10-isopropyl-9-(trimethylsilyl)-9,10-dihydroanthracene (S4)

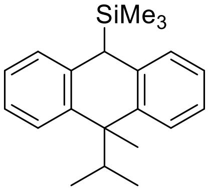

S4

10-Methyl-10-(trimethylsilyl)-9,10-dihydroanthracene (**S3**, 7.94 g, 29.6 mmol) was dissolved in THF (150 mL, 0.2 M), under inert atmosphere and the reaction mixture was cooled down to 0 °C. *n*-Butyllithium (22.2 mL, 1.63 M in hexane, 35.5 mmol) was added dropwise and the reaction mixture was stirred for 2 h. Subsequently, *iso*-propyl iodide (4.40 mL, 44.4 mmol) was added and the mixture was left stirring for 30 min. The solution was allowed to warm to room temperature and then kept stirring for 1 h. Upon completion, the product was extracted with diethyl ether (3 x 100 mL). The combined organic layer was washed once with water (100 mL) and once with brine (100 mL). The solution was then dried over $MgSO_4$ and filtered over a silica pad. After the removal of the solvent under reduced pressure, recrystallization from ethanol (100 mL) afforded the product as white crystals (6.90 g, 22.4 mmol, 76% yield). The data is in accordance with the literature.[2]

^{1}H NMR (400 MHz, $CDCl_3$) δ 7.40 (dt, *J* = 7.5, 2.8 Hz, 2H), 7.15 – 7.06 (m, 4H), 7.00 – 6.93 (m, 2H), 3.84 (s, 1H), 1.98 (p, *J* = 6.8 Hz, 1H), 1.85 (s, 3H), 0.53 (d, *J* = 6.9 Hz, 6H), -0.23 (s, 9H).
^{13}C{^{1}H} NMR (101 MHz, $CDCl_3$) δ 139.5, 135.7, 127.2, 127.1, 125.0, 124.4, 45.3, 45.0, 39.5, 27.6, 18.8, -1.7.
HRMS (APCI pos) calcd. $C_{21}H_{29}Si$ $[M+H]^+$ 309.2033, found 309.2034.

10-Methyl-10-isopropyl-9,10-dihydroanthracene (S5)

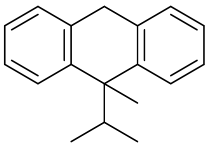

S5

Tetra-*n*-butyl ammonium fluoride (44.5 mL, 1 M in THF, 44.5 mmol) was added to a solution of 10-methyl-10-isopropyl-9-(trimethylsilyl)-9,10-dihydroanthracene (**S4**, 6.90 g, 22.23 mmol) dissolved in THF (110 mL, 0.2 M). The reaction mixture was kept stirring for 1 h at room temperature. Upon completion , the mixture was washed with water (100 mL), extracted with diethyl ether (100 mL) and then dried over $MgSO_4$ and filtered over a silica pad. The solvent

was removed under reduced repssure. The product was obtained as white crystals (3.77 g, 15.8 mmol, 71% yield) through recrystallization from methanol (80 mL). The data is in accordance with the literature.[2]

^{1}H NMR (400 MHz, cdcl$_3$) δ 7.46 (d, *J* = 7.7 Hz, 2H), 7.30 – 7.14 (m, 6H), 4.23(d, *J* = 18.7 Hz, 1H), 3.90 (d, *J* = 18.7 Hz, 1H), 1.95 (p, *J* = 6.9 Hz, 1H), 1.72 (s, 3H), 0.68 (d, *J* = 6.8 Hz, 6H).
^{13}C{^{1}H} NMR (101 MHz, cdcl$_3$) δ 143.9, 136.4, 128.0, 126.5, 125.8, 125.8, 46.3, 36.7, 35.5, 18.5, 17.8.
GC-MS (EI) calcd. $C_{18}H_{20}$ [M] 236.36, found 193 (M- C_3H_7), 178 (M- C_4H_{10}).

10-Methyl-10-isopropyl-anthracen-9-one (S6)

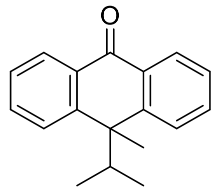

S6

Potassium permanganate (7.50 g, 47.5 mmol) and active manganese dioxide (22.5 g, 259 mmol) were grinded to a homogenous powder. The oxidant mixture was added portionwise over 15 min to a solution of 10-methyl-10-isopropyl-9,10-dihydroanthracene (**S5**, 3.00 g, 12.59 mmol) in dichloromethane (190 mL, 0.066 M). The reaction mixture was kept at room temperature under vigorous stirring for 6 h. Upon completion, the mixture was filtered over a celite pad. The resulting product was obtained as white crystals (3.10 g, 12.1 mmol, 96% yield). The data is in accordance with the literature.[2]

^{1}H NMR (400 MHz, CDCl$_3$) δ 8.34 (d, *J* = 7.8 Hz, 2H), 7.68 – 7.57 (m, 4H), 7.44 (ddd, *J* = 8.1, 6.5, 1.8 Hz, 2H), 2.07 (hept, *J* = 6.9 Hz, 1H), 1.90 (d, *J* = 1.7 Hz, 3H), 0.62 (d, *J* = 6.7 Hz, 6H).
^{13}C{^{1}H} NMR (101 MHz, CDCl$_3$) δ 185.0, 147.9, 132.7, 132.6, 127.2, 127.2, 126.8, 45.1, 43.4, 24.9, 18.4.
HRMS (APCI pos) calcd. $C_{18}H_{19}O$ $[M+H]^+$ 251.1430, found 251.1430.

10-Methyl-10-isopropyl-anthracen-9-thione (3)

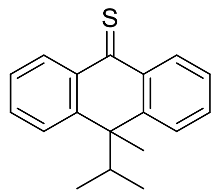

3

10-Methyl-10-isopropyl-anthracen-9-one (**S6**, 200 mg, 800 µmol) was dissolved in toluene (4 mL, 0.2 M) under inert atmosphere. P_2S_5 (750 mg, 3.38 mmol) was added and the reaction mixture was heated to 90 °C for 16 h. The solvent was removed under reduced pressure and the crude product was immediately submitted to flash column chromatography (SiO_2, 100% pentane). The purified product was obtained as a blue oil (188 mg, 705 µmol, 88% yield).

Note: The product should be used within a few days due to reduced stability.

^{1}H NMR (400 MHz, C_6D_6) δ 8.79 (d, *J* = 8.0 Hz, 2H), 7.25 – 7.14 (m, 4H), 7.03 (ddd, *J* = 8.3, 5.6, 2.7 Hz, 2H), 1.69 (hept, *J* = 6.8 Hz, 1H), 1.47 (s, 3H), 0.43 (d, *J* = 6.8 Hz, 6H).
^{13}C{^{1}H} NMR (101 MHz, C_6D_6) δ 142.2, 141.3, 131.8, 130.1, 126.9, 126.4, 46.1, 44.3, 21.5, 18.4.

10-Isopropyl-2,10-dimethyl-2,3-dihydro-10*H*-dispiro[anthracene-9,2-thiirane-3,1-benzo[*f*]thiochromene] (5)

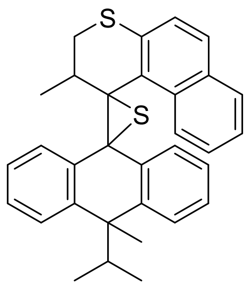

5

(2-Methyl-2,3-dihydro-1H-benzo[*f*]thiochromen-1-ylidene)-hydrazine[3] (**4**, 201 mg, 837 µmol) was dissolved in *N*,*N*-dimethylformamide (2.0 mL, 0.42 M). The reaction mixture was cooled down to -50 °C and a solution of (diacetoxy)iodobenzene (184 mg, 572 µmol) in *N*,*N*-dimethylformamide (2.0 mL) was added. After 1 min, a solution of 10-methyl-10-isopropyl-anthracen-9-thione (**3**, 149 mg, 558 µmol) in THF (2.0 mL, 0.29 M) was added dropwise. The reaction mixture was left stirring at -50 °C for 1 h before allowing it to warm to room temperature and stirring was continued for 16 h. Upon completion, the obtained mixture was diluted with ethyl acetate (100 mL) and a saturated aqueous NH_4Cl solution. The phases were separated and the aqueous layer was extracted three times with ethyl acetate (20 mL). The combined organic layer was then washed with water (2 x 50 mL), brine (2 x 100 mL), dried over Na_2SO_4 and filtered over a silica pad. The product was obtained through flash column chromatography (pentane:dichloromethane 2:1) as white crystals as a mixture of diastereoisomers in a 1:1 ratio (148 mg, 309 µmol, 55% yield).

Note: The isomers were not separated and were used as such for NMR analysis.

^{1}H NMR (400 MHz, $CDCl_3$) δ 9.50 (dd, *J* = 8.9, 1.1 Hz, 1H), 8.80 (dd, *J* = 8.8, 1.2 Hz, 1H), 8.08 (dd, *J* = 7.9, 1.5 Hz, 1H), 8.02 (dd, *J* = 7.7, 1.6 Hz, 1H), 7.72 (dd, *J* = 8.2, 1.4 Hz, 1H), 7.57 (ddd, *J* = 7.5, 6.2, 1.4 Hz, 3H), 7.54 – 7.42 (m, 2H), 7.42 – 7.23 (m, 8H), 7.20 (dd, *J* = 8.0, 1.4 Hz, 1H), 7.14 (dd, *J* = 7.9, 1.3 Hz, 1H), 6.87 – 6.75 (m, 4H), 6.48 (dd, *J* = 8.1, 1.4 Hz, 1H), 6.36 (ddd, *J* = 8.3, 7.1, 1.3 Hz, 1H), 6.03 (ddd, *J* = 8.2, 7.0, 1.3 Hz, 1H), 3.13 (ddt, *J* = 11.4, 9.7, 5.7 Hz, 1H), 2.82 (qdd, *J* = 6.9, 4.7, 2.7 Hz, 1H), 2.47 (dd, *J* = 12.5, 4.7 Hz, 1H), 2.40 (dd, *J* = 12.5, 2.7 Hz, 1H), 2.17 (s, 3H), 2.16 – 2.05 (m, 2H), 1.96 – 1.81 (m, 2H), 1.63 (s, 3H), 1.39 (d, *J* = 6.8 Hz, 3H), 1.01 (d, *J* = 6.9 Hz, 3H), 0.91 (d, *J* = 6.8 Hz, 4H), 0.44 – 0.36 (m, 9H).

^{13}C{^{1}H} NMR 101 MHz, $CDCl_3$) δ 147.7, 145.9, 145.5, 143.3, 137.6, 136.2, 135.3, 135.2, 134.0, 133.1, 133.0, 132.4, 131.8, 131.7, 131.6, 131.4, 130.0, 128.7, 128.6, 128.0, 127.3, 127.2, 127.1, 127.03, 127.01, 126.9, 126.3, 126.0, 125.8, 125.7, 125.6, 125.5, 125.2, 124.9, 124.6,

124.43, 124.35, 124.0, 123.9, 123.8, 123.1, 122.8, 122.2, 64.7, 63.1, 62.3, 57.5, 46.0, 45.9, 45.5, 40.1, 37.0, 36.7, 34.8, 31.7, 26.8, 21.5, 18.7, 18.5, 18.4, 17.9, 17.2, 15.9, 14.2.
HRMS (APCI pos) calcd. $C_{32}H_{31}S_2$ $[M+H]^+$ 479.1862, found 479.1856.

(10-Isopropyl-10-methylanthracen-9(10*H*)-ylidene)-2-methyl-2,3-dihydro-1*H*-benzo[*f*]thiochromene (2)

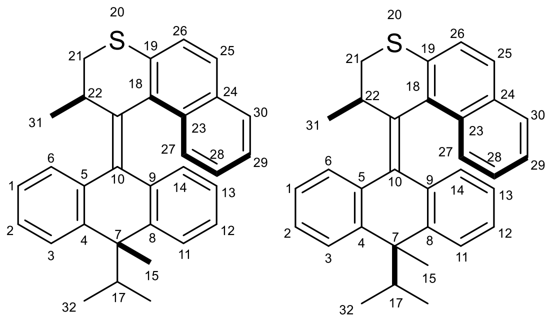

(*S*a)-**2S** (*R*a)-**2S**

10-Isopropyl-2,10-dimethyl-2,3-dihydro-10H-dispiro[anthracene-9,2-thiirane-3,1-benzo[*f*]thiochromene] (**5**, 148 mg, 304 µmol) was dissolved in toluene (3 mL, 0.1 M) and hexaethyl phosphorus triamide (0.1 mL, 608 µmol) was added to the solution. The reaction mixture was kept under stirring at 80 °C for 16 h. The reaction mixture was concentrated under reduced pressure and the crude product was purified by flash column chromatography (pentane-dichloromethane- 95:5). Two pure diastereomers were obtained in separate fractions ((*S*a)-**2**: 78 mg, 175 µmol, 57% and (*R*a)-**2**: 55 mg, 123 µmol, 41%, in total 133 mg, 298 mmol, 98%).

(*S*a)-**2S**

^{1}H NMR (600 MHz, $CDCl_3$) δ 8.31 (d, *J* = 8.4 Hz, 1H, H-27), 7.88 (d, *J* = 8.2 Hz, 1H, H-30), 7.74 – 7.71 (m, 1H, H-1), 7.68 (d, *J* = 8.5 Hz, 1H, H-25), 7.60 (dd, *J* = 7.7 Hz, 1H, H-28), 7.53 – 7.48 (m, 2H, H-29 + H-3), 7.37 (d, *J* = 8.5 Hz, 1H, H-26), 7.33 – 7.31 (m, 2H, H-2 + H-6), 7.27 (d, *J* = 7.6 Hz, 1H, H-11), 6.92 (dd, *J* = 7.6, 7.6 Hz, 1H, H-12), 6.88 (d, *J* = 7.9 Hz, 1H, H-14), 6.49 (dd, *J* = 7.6, 7.6 Hz, 1H, H-13), 4.29 – 4.20 (m, 1H, H-22), 2.96 (A of AB, dd, *J* = 11.7, 8.8 Hz, 1H, H-21), 2.83 (hept, *J* = 6.6 Hz, 1H, H-17), 2.50 (B of AB, dd, *J* = 11.8, 4.7 Hz, 1H, H-21), 1.64 (s, 3H, H-15), 1.06 (d, *J* = 6.8 Hz, 3H, H-31), 0.73 (A of AB, d, *J* = 6.9 Hz, 3H, H-32), 0.62 (B of AB, d, *J* = 6.7 Hz, 3H, H-32).

^{13}C{^{1}H} NMR (151 MHz, $CDCl_3$) δ 146.6 (C_{quat}), 144.3 (C_{quat}), 138.7 (C_{quat}), 137.0 (C_{quat}), 136.6 (C_{quat}), 136.3 (C_{quat}), 135.6 (C_{quat}), 135.4 (C_{quat}), 135.3 (C_{quat}), 131.6 (C_{quat}), 128.85 (CH_{Ar}), 128.82 (CH_{Ar}), 127.9 (CH_{Ar}), 127.3 (CH_{Ar}), 127.1 (CH_{Ar}), 126.8 (CH_{Ar}), 125.9 (CH_{Ar}), 125.6

(CH_{Ar}), 125.5 (CH_{Ar}), 125.3 (CH_{Ar}), 124.9 (CH_{Ar}), 124.8 (CH_{Ar}), 47.3 (C-7), 39.9 (C-22), 37.3 (C-21), 34.8 (C-16), 21.8 (C-31), 18.6 (C-32), 18.4 (C-32), 14.6 (C-15).

(R_a)-$\mathbf{2_S}$

^{1}H NMR (600 MHz, $CDCl_3$) δ 7.75 (dd, J = 5.7, 3.4 Hz, 1H, H-1), 7.73 (d, J = 8.5 Hz, 1H, H-25), 7.70 (d, J = 8.1 Hz, 1H, H-6), 7.57 (d, J = 8.4 Hz, 1H, H-26), 7.55 – 7.53 (m, 1H, H-3), 7.42 (d, J = 8.4 Hz, 1H, H-27), 7.33 – 7.31 (m, 2H, H-2 + H-11), 7.29 (d, J = 8.8 Hz, 1H, H-30), 7.20 (dd, J = 7.4, 7.4 Hz, 1H, H-29), 6.97 (dd, J = 8.6, 6.7 Hz, 1H, H-28), 6.82 (dd, J = 7.6, 1.4 Hz, 1H, H-12), 6.31 (dd, J = 7.5, 7.5 Hz, 1H, H-13), 6.14 (d, J = 7.7 Hz, 1H, H-14), 4.56 – 4.50 (m, 1H, H-22), 3.68 (A of AB, dd, J = 11.8, 9.0 Hz, 1H, H-21), 2.96 (B of AB, dd, J = 11.8, 4.1 Hz, 1H, H-21), 2.83 (hept, J = 6.7 Hz, 1H, H-17), 1.71 (s, 3H, H-15), 1.13 (A of AB, d, J = 6.8 Hz, 3H, H-32), 0.97 (B of AB, d, J = 6.7 Hz, 3H, H-32), 0.84 (d, J = 6.8 Hz, 3H, H-31).

^{13}C{^{1}H} NMR (151 MHz, $CDCl_3$) δ 146.9 (C_{quat}), 144.0 (C_{quat}), 137.6 (C_{quat}), 137.4 (C_{quat}), 135.8 (C_{quat}), 135.3 (C_{quat}), 134.6 (C_{quat}), 132.8 (C_{quat}), 132.3 (C_{quat}), 131.6 (C_{quat}), 128.5 (CH_{Ar}), 128.0 (CH_{Ar}), 127.44 (CH_{Ar}), 127.38 (CH_{Ar}), 127.33 (CH_{Ar}), 126.5 (CH_{Ar}), 126.0 (CH_{Ar}), 125.9 (CH_{Ar}), 125.60 (CH_{Ar}), 125.58 (CH_{Ar}), 125.4 (CH_{Ar}), 125.2 (CH_{Ar}), 124.8 (CH_{Ar}), 124.4 (CH_{Ar}), 47.7 (C-7), 37.5 (C-21), 35.8 (C-22), 35.2 (C-16), 21.0 (C-31), 19.0 (C-32), 18.8 (C-32), 14.7 (C-15).

HRMS (APCI pos) calcd. $C_{32}H_{31}S_2$ $[M+H]^+$ 447.2141, found 447.2136.

Note on the stereochemical elements of the different diastereomers of motor 2:

Additionally, to the molecule numbering (**2**), we indicate the axial chirality of the molecule through the nominator S_a or R_a. The subscript **M** or **S** as in $\mathbf{2_S}$ and $\mathbf{2_M}$ indicate if the isomer is thermally stable (**S**) or metastable (**M**). Only when talking about enantiomers separated by CD do we extend the descriptors *P* and *M* to specify the helicity of the molecule.

Upon synthesis of the motor two diastereomers are isolated as a racemic mixture (two diastereomers of two enantiomers each, 4 isomers in total): (*S*)-(*P*)-(S_a)-$\mathbf{2_S}$, (*R*)-(*M*)-(S_a)-$\mathbf{2_S}$, (*S*)-(*P*)-(R_a)-$\mathbf{2_S}$ and (*R*)-(*M*)-(R_a)-$\mathbf{2_S}$. The sets of two motors with the same axial chirality S_a and R_a were separated on flash column chromatography ((*S*)-(*P*)-(S_a)-$\mathbf{2_S}$ and (*R*)-(*M*)-(S_a)-$\mathbf{2_S}$ from (*S*)-(*P*)-(R_a)-$\mathbf{2_S}$ and (*R*)-(*M*)-(R_a)-$\mathbf{2_S}$). The enantiomers of opposite chirality of the S_a motors ((*S*)-(*P*)-(S_a)-$\mathbf{2_S}$ and (*R*)-(*M*)-(S_a)-$\mathbf{2_S}$,) were separated on SFC and CD spectra were recorded. Their point chiral center also is opposite, as for this motor scaffold the upper half methyl group of the *M* stable isomer always adopts the *R* configuration and the *P* isomer always adopts the *S* configuration, in order to be in the preferred *pseudo* axial position.

Photochemical isomerization inverts the stereochemistry of the molecule. In total, motor **2** contains three elements of chirality. The point chiral center located in the upper half of the molecule is fixed and hence does not change even when the molecule is performing rotary motion. The helicity of the molecule interconverts during the rotational movement. A motor isolated in its stable form as the *M* isomer, will form a metastable isomer in *P* helicity and *vice versa*. The element of axial chirality inverts between the stable isomers, so only after performing the thermal/photochemical helix inversion.

X-ray crystal structure of (R_a)-$\mathbf{2_S}$

Single-crystals of (R_a)-$\mathbf{2_S}$ were obtained by slow diffusion of pentane into a concentrated solution in dichloromethane. The crystal was mounted on a cryoloop and placed in the nitrogen stream (100 K) of a Bruker-AXS D8 Venture diffractometer. Data collection and processing was carried out using the Bruker APEX3 software (Bruker, 2016. APEX3 (V2016.1-0), SAINT (Version 8.37A) and SADABS (Version 2014/15). Bruker AXS Inc., Madison, Wisconsin, USA). A multi-scan absorption correction was applied, based on the intensities of symmetry-related reflections measured at different angular settings (SADABS).[4] The structure was solved using SHELXT[5] and refinement was performed using SHELXL.[6] The hydrogen atoms were generated by geometrical considerations, constrained by idealised geometries and allowed to ride on their carrier atoms with an isotropic displacement parameter related to the equivalent displacement parameter of their carrier atoms. No A- or B-level alerts were raised by CheckCIF for the fully refined structures. A root mean squares deviation was calculated between the X-ray structures and their computed structures (DFT, ωB97X-D/def2-SVP, gas phase).

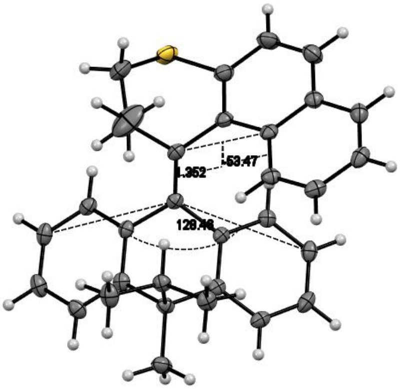

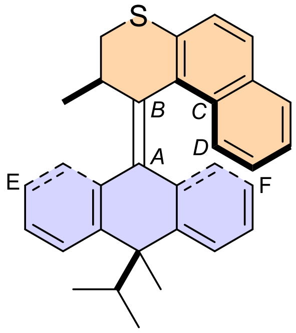

(R_a)-$\mathbf{2_S}$

C=C length / Å (*AB*)	Dihedral angle / ° (*ABCD)*	Folding angle / ° (*EAF)*
1.352	53.47	128.46

Figure S1: *Left: ORTEP image (ellipsoid probability at 50%) of the X-ray structure of (R_a)-$\mathbf{2_S}$, with key bond lengths, angles and torsions highlighted. Right: Labelling of (R_a)-$\mathbf{2_S}$.*

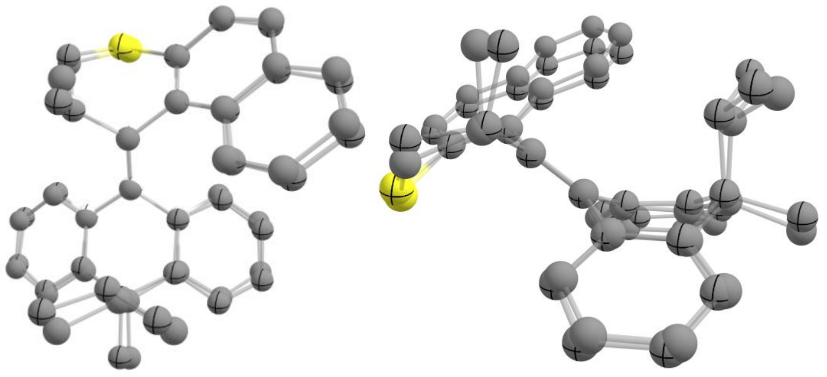

Figure S2: *X-ray structures of (R$_a$)-**2$_S$** with their optimised structures (DFT, B3LYP/6-31G(d,p)) overlaid. The experimentally determined structure has grids on the atoms, the DFT simulated structure has no grids on the atoms.*

Table S1: *Crystal data and structure refinement for (R_a)-**2s**.*

Empirical formula	$C_{32}H_{30}S$
Formula weight	446.62
Temperature/K	100.0
Crystal system	monoclinic
Space group	$P2_1/c$
a/Å	16.4882(5)
b/Å	17.3137(5)
c/Å	8.5841(3)
α/°	90
β/°	92.5590(10)
γ/°	90
Volume/Å^3	2448.07(13)
Z	4
ρ_{calc}g/cm^3	1.212
μ/mm^{-1}	1.285
F(000)	952.0
Crystal size/mm^3	0.342 × 0.223 × 0.111
Radiation	CuKα (λ = 1.54178)
2Θ range for data collection/°	5.364 to 136.398
Index ranges	-19 ≤ h ≤ 19, -20 ≤ k ≤ 20, -10 ≤ l
Reflections collected	49299
Independent reflections	4476 [R_{int} = 0.0398, R_{sigma} =
Data/restraints/parameters	4476/0/303
Goodness-of-fit on F^2	1.046
Final R indexes [I>=2σ (I)]	R_1 = 0.0367, wR_2 = 0.0984
Final R indexes [all data]	R_1 = 0.0400, wR_2 = 0.1008
Largest diff. peak/hole / e Å^{-3}	0.74/-0.32

Photochemical and Thermal Isomerisation Studies

General procedures

All photochemical and thermal studies were carried out using a Varian Unity Plus (500 MHz) NMR spectrometer. An NMR tube containing 300 µL of a 2 mM solution of (S_a)-$\mathbf{2}_S$ or (R_a)-$\mathbf{2}_S$ respectively was fitted with coaxial tube accommodating a glass fibre optic cable connected to a 365 nm LED (ThorLabs, M365FP1). An initial spectrum was recorded at a given temperature before switching on the connected LED set to half power for *in situ* irradiation. Irradiation was continued until the maximum amount of metastable state was obtained. Next, the sample was removed and the spectrometer probe was warmed to an appropriate temperature for thermal helix inversion (55 °C to 75 °C for (R_a)-$\mathbf{2}_M$ and 0 °C to 20 °C for (S_a)-$\mathbf{2}_M$). The sample was reinserted after the temperature had stabilised and the thermal decay could be followed by consecutive measuring.

The obtained NMR spectra arrays were processed (phase and baseline correction) and normalized to 100 using a signal, which remained unchanged during thermal decay of the metastable state (Figure S5 and S7) or over the course of the photochemically induced isomerizations (figure 4 in the main text and Figure S8).

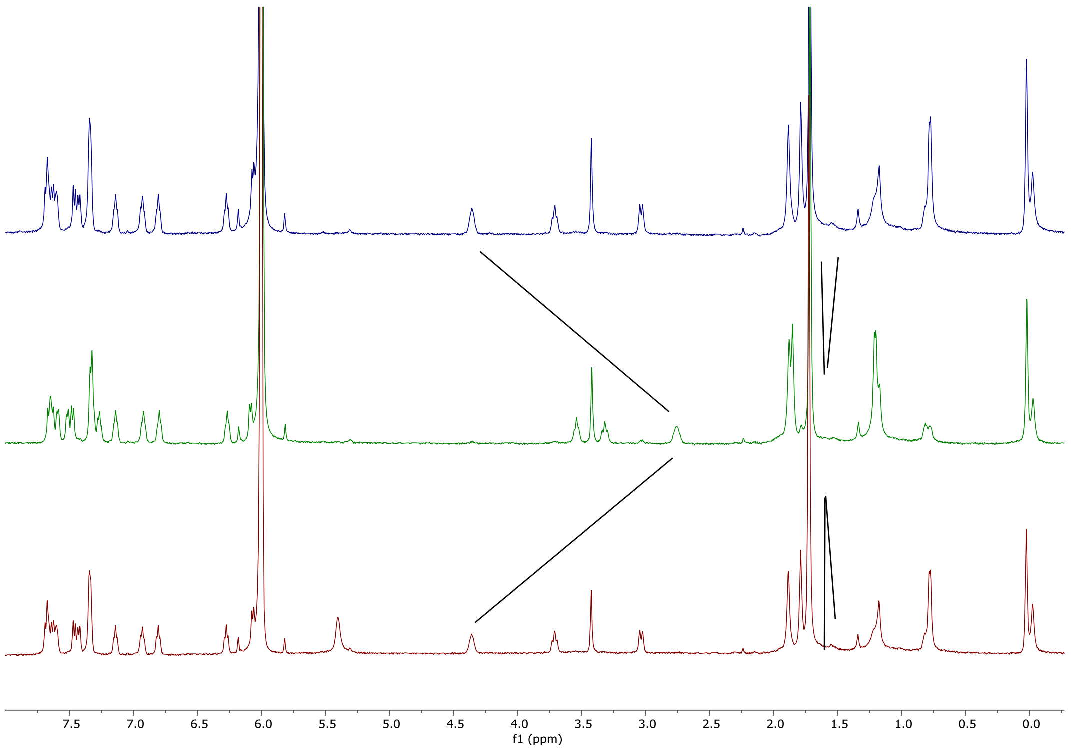

Figure S3: *Motor **1** in tetrachloroethane-d_2 at -35 °C (2 mM) (blue, top), irradiation with 365 nm to the photostationary state (97:3-metastable:stable) at -35 °C (green, middle), after thermal helix inversion at 55 °C (red, bottom).*

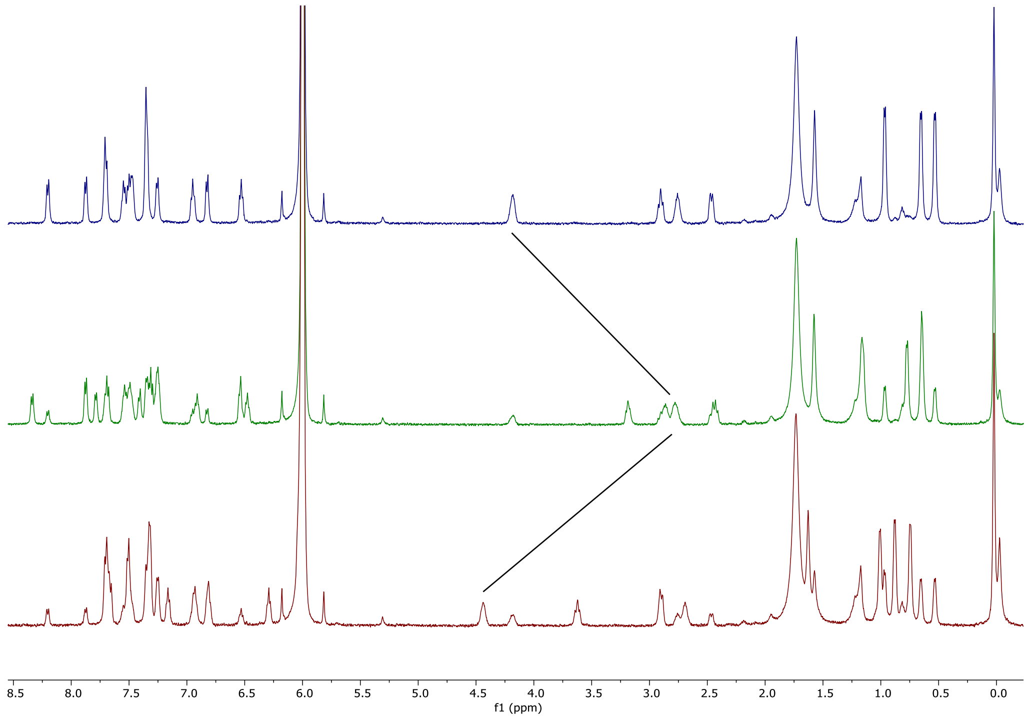

Figure S4: *Full ^{1}H-NMR spectra of isomer (S_a)-**2_S** in tetrachloroethane-d_2 (2mM) at -35 °C initial (blue, top), after 1 h irradiation with 365 nm LED (green, middle) establishing a photostationary state ratio of 68:32 ((S_a)-**2_M** : (S_a)-**2_S**). (R_a)-**2_S** forms in a molar ratio of 68:32 ((R_a)-**2_S** : (S_a)-**2_S** after keeping the sample at 20 °C overnight) (red, bottom).*

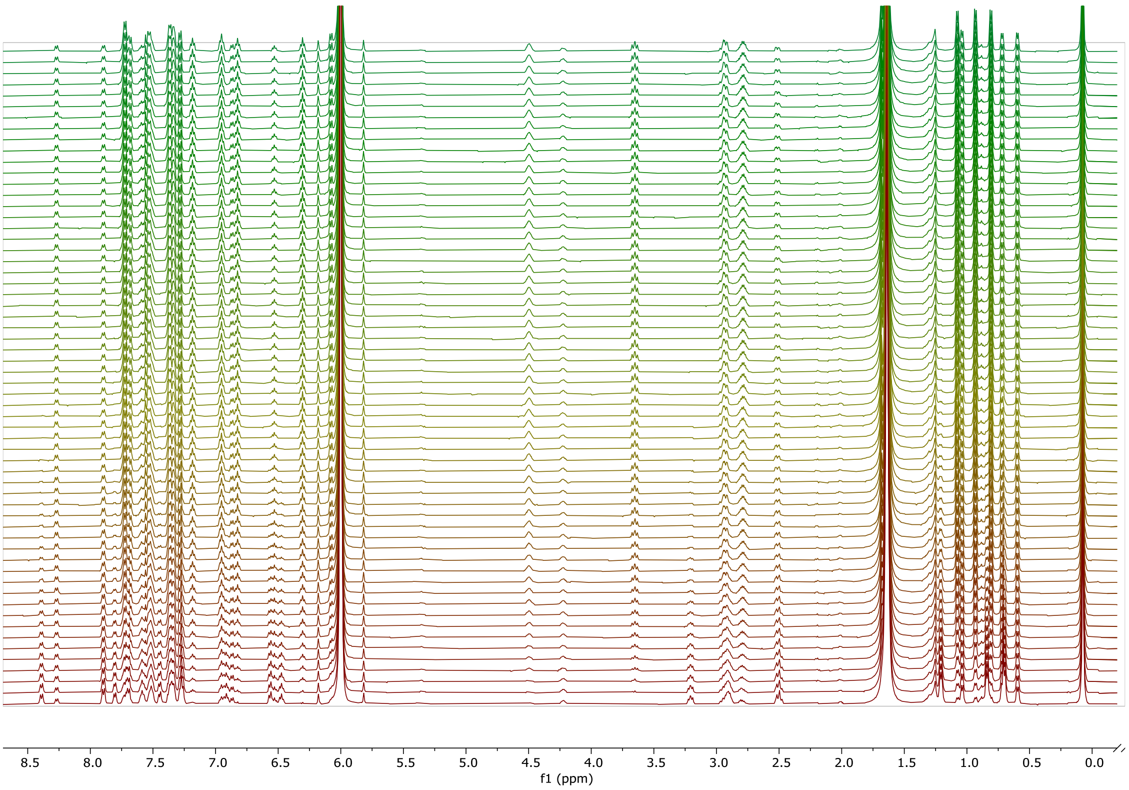

Figure S5: *^{1}H-NMR spectra of isomer (S$_a$)-**2$_S$** in tetrachloroethane-d$_2$ (2mM). From bottom to top: decay of metastable state (S$_a$)-**2$_M$** and formation of (R$_a$)-**2$_S$** at 20 °C over the course of 5 h and measuring every 5 min.*

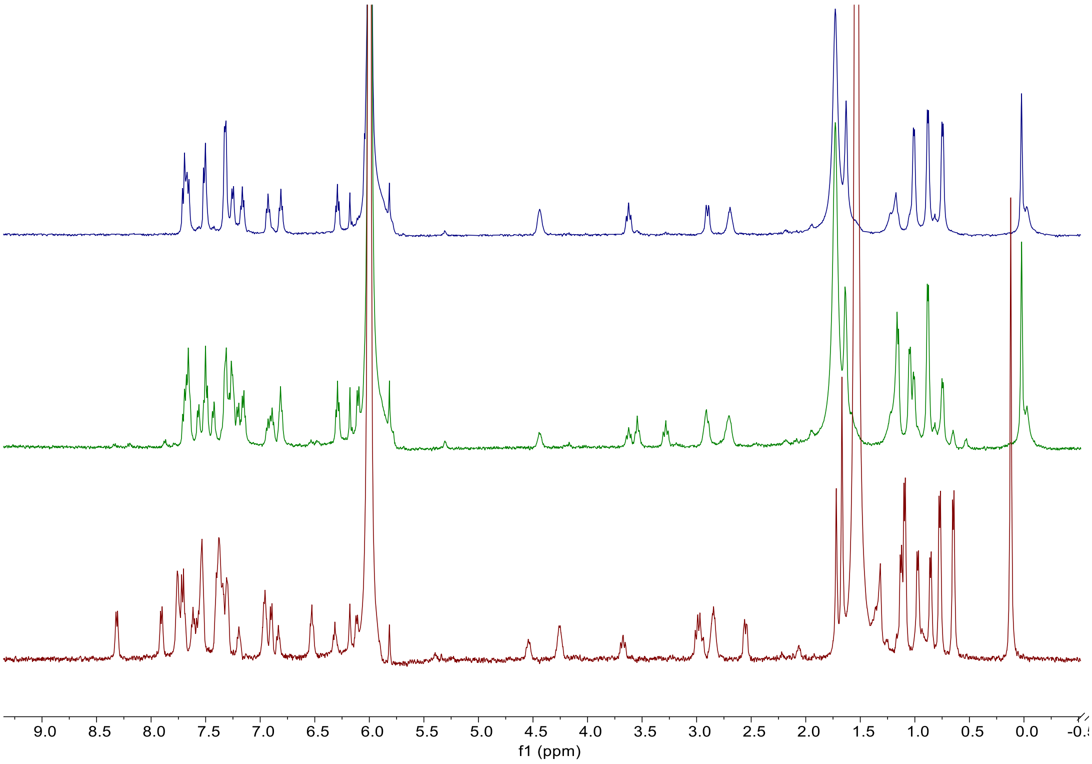

Figure S6: *^{1}H-NMR spectra of isomer (R$_a$)-**2$_S$** in tetrachloroethane-d$_2$ (2mM) at -35 °C initial (blue, top), after 30 min. irradiation at 365 nm (green, middle) and after maintaining at 75 °C overnight (red, bottom).*

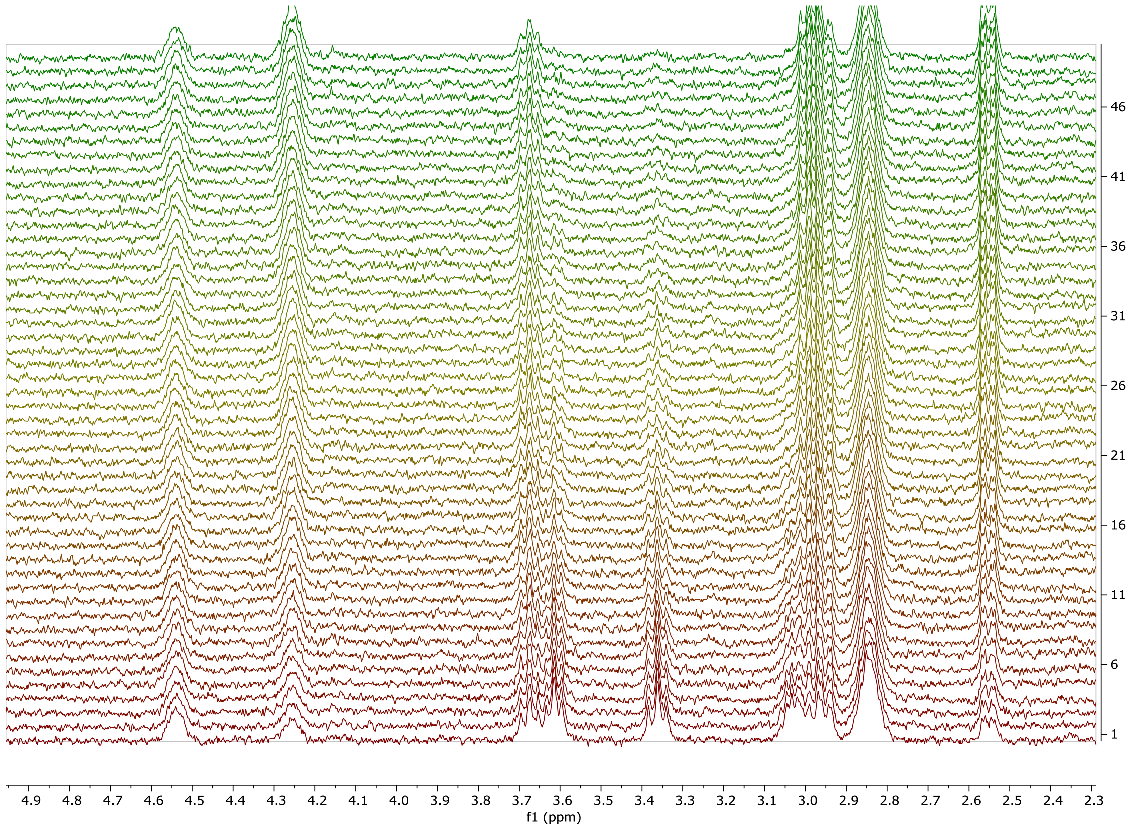

Figure S7: *^{1}H-NMR spectra of isomer (R$_a$)-**2$_M$** in tetrachloroethane-d$_2$ (2mM) at 75 °C. From bottom to top: decay of metastable state (R$_a$)-**2$_M$** and formation of (S$_a$)-**2$_S$**.*

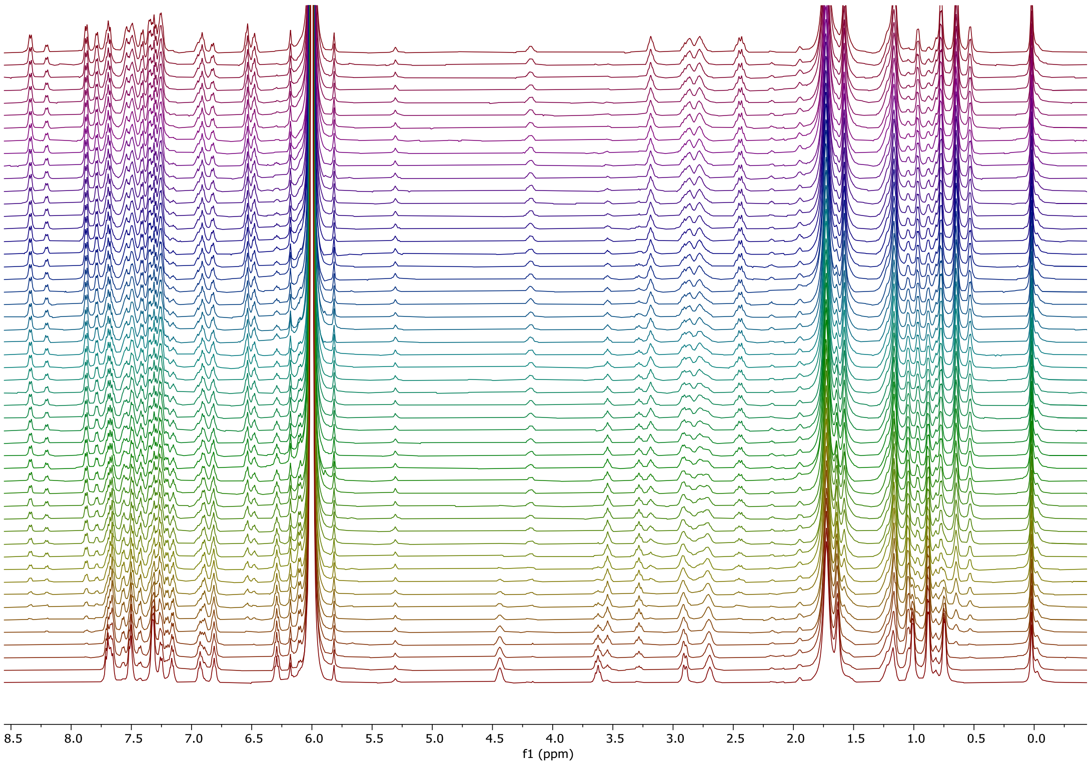

Figure S8: *From bottom to top: ^{1}H-NMR spectra Irradiation of isomer (R$_a$)-**2$_S$** in tetrachloroethane-d$_2$ (2mM) for 3 h, starting from the bottom spectrum, a first metastable state (R$_a$)-**2$_M$** forms, which is photochemically transformed in to isomer (S$_a$)-**2$_S$**, which then forms a metastable state (S$_a$)-**2$_M$**, ending at the same PSS as irradiation starting from isomer (S$_a$)-**2$_S$**.*

Eyring Analysis

General Procedures

Eyring plot analysis was performed to determine the Gibbs free energy and half-life of the thermal decay of (R_a)-**2_M** and (S_a)-**2_M**. In most instances, the decrease in absorption in a UV/Vis spectrum is observed over time. However, in this case, largely overlapping UV/Vis absorption spectra do not allow the assignment of absorption bands to each isomer. Furthermore, the photochemical helix inversion of (R_a)-**2_M** impedes gaining information on the molar ratio of each isomer in the mixture after irradiation. In this case, we decided to perform Eyring analysis on kinetic data obtained by ^{1}H NMR measurements, as it allows to clearly follow the decay of each isomer through observing the decay of well separated signals in ^{1}H-NMR. As both thermal helix inversion processes happen at vastly different temperatures, they were observed in separate measurements.

A solution of (R_a)-**2_S** in toluene-d_8 (2 mM) was filled in an NMR tube fitted with a coaxial tube accommodating a fibre optic cable connected to a 365 nm LED by Thorlabs (M365F1) for *in situ* irradiation in the NMR tube. First, the sample was irradiated in an NMR spectrometer (300 MHz probe) at 0 °C. The photokinetics were monitored by continuous recording of spectra. To enrich the maximum molar ratio of (R_a)-**2_M** the sample was irradiated for 5 min, and to reach the maximum amount of (S_a)-**2_M**, the sample was irradiated for 1 h. A different NMR spectrometer (Varian Unity Plus, 500 MHz) was brought to the appropriate temperature to observe THI of either (R_a)-**2_M** (57, 62, 67, 72 or 77 °C) or (S_a)-**2_M** (-3, 2, 7, 12, 17 °C) and left to stabilise for around 1 h. The temperature was confirmed by temperature calibration with a sample of diethylene glycol or methanol prior to and after each measurement. The samples were moved to the spectrometer at the appropriate temperature for thermal helix inversion to complete within a few hours immediately after irradiation and equilibrated for around 5 minutes. After tuning, locking, and shimming the instrument, the thermal decay of the respective metastable compounds was followed by measuring ^{1}H NMR spectra every 5-20 minutes depending on the time needed for the process to complete.

The rate constant of the first order decay (*k*) is obtained at five different temperatures by fitting the y = A1*exp(-x/t1) + y0 equation using the Origin software. A least square analysis on the Eyring equation was performed to obtain the Gibbs free energy of the thermal helix inversion.

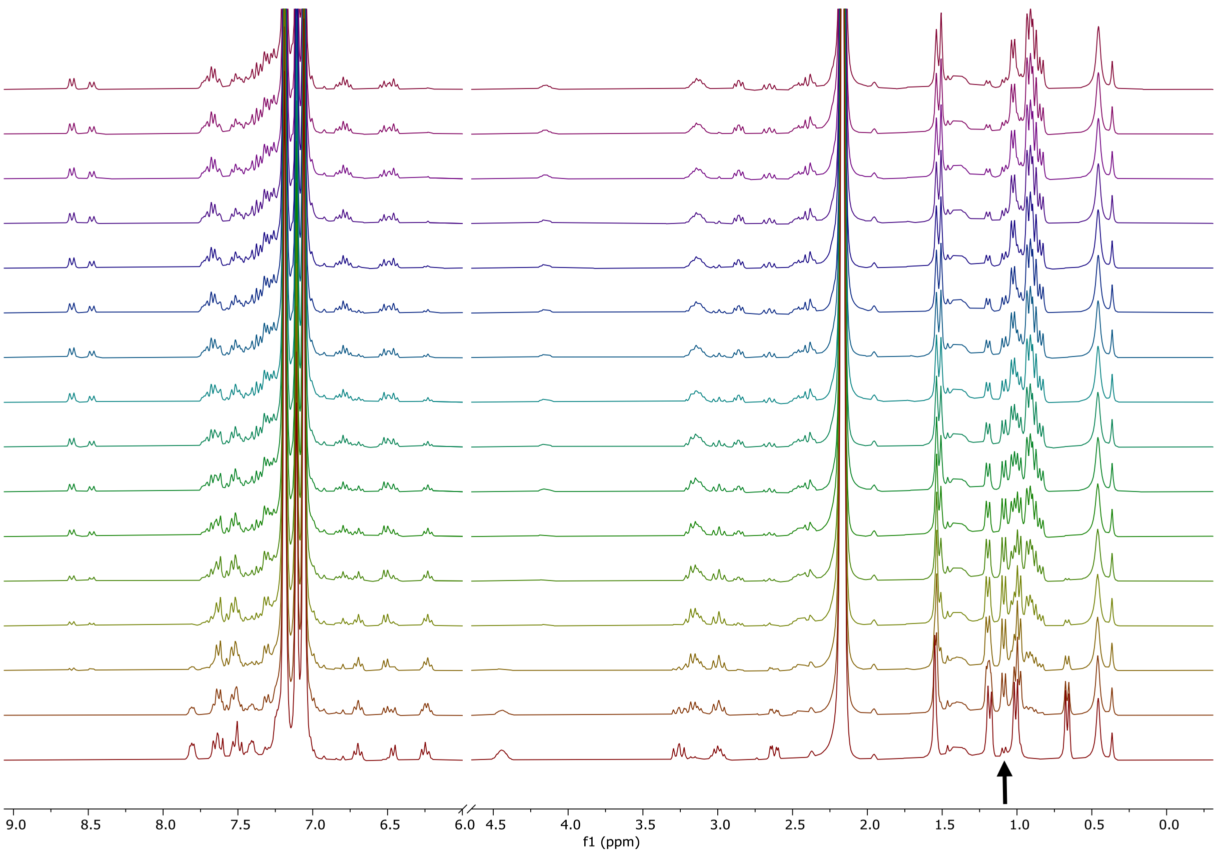

Figure S9: *Irradiation of (R$_a$)-**2$_S$** at 365 nm in toluene-d$_8$ at 0 °C. Spectra are recorded every 4 min. The formation and decay of (R$_a$)-**2$_M$** can be followed by observing the signal at 1.08 ppm. To enhance the amount (R$_a$)-**2$_M$** molar ratio in solution the irradiation was stopped after 5 min.*

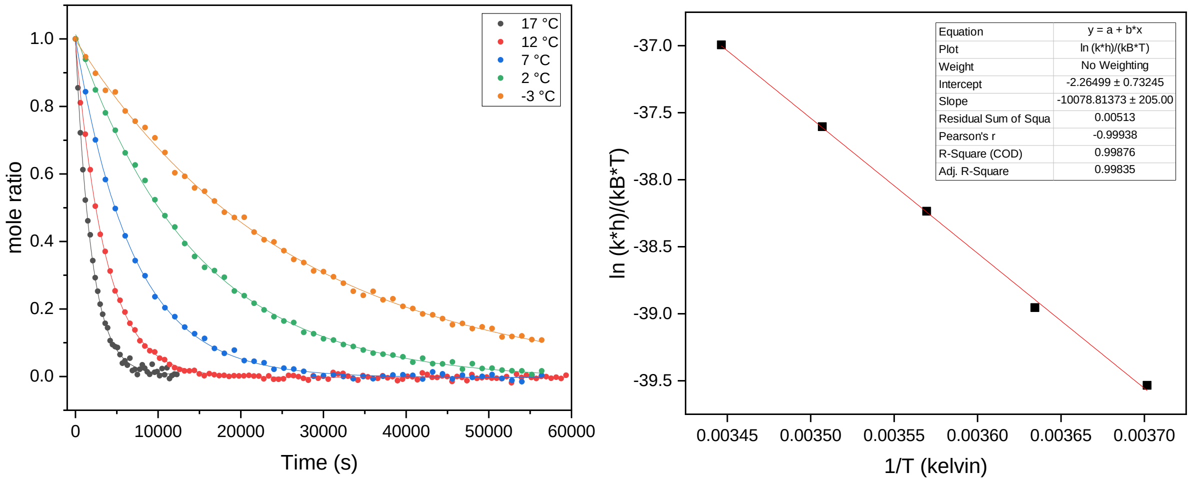

Figure S10: *The decay of the signal at 8.52 ppm in the* ^{1}*H-NMR spectrum of (S*$_a$*)-**2**$_M$ *in toluene-d*$_8$ *was observed at the given temperatures (left). Eyring plot of the thermal decay of (S*$_a$*)-**2**$_M$ *to (R*$_a$*)-**2**$_S$*.*

Table S2: *Activation parameters of (S*$_a$*)-**2**$_M$ *to (R*$_a$*)-**2**$_S$ *at 20 °C.*

**$\Delta G^{\ddagger}_{293\,K}$ (kJ/mol)**	**$\Delta G^{\ddagger}_{293\,K}$ (kcal/mol)**	**Half life (293 K)**
89.3 ± 0.3	21.3 ± 0.1	0.26 h
**$\Delta H^{\ddagger}_{293\,K}$ (kcal/mol)**	**$\Delta H^{\ddagger}_{293\,K}$ (kJ/mol)**	
83.8	20.0	
**$\Delta S^{\ddagger}_{293\,K}$ (kcal/mol)**	**$\Delta S^{\ddagger}_{293\,K}$ (kJ/mol)**	
-18.8	-4.4	

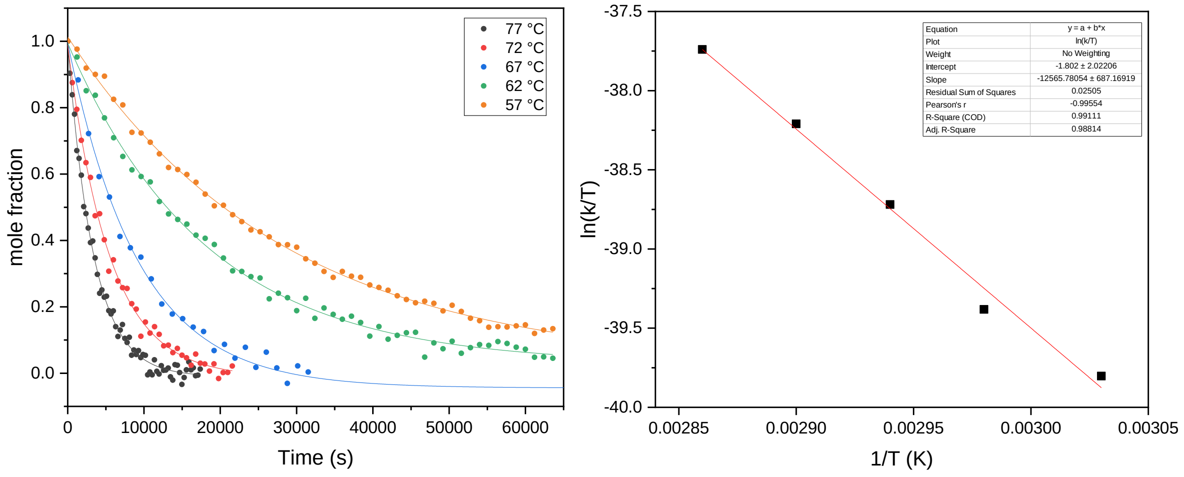

Figure S11: *Monitoring the decay of the signal at 3.18 ppm in the ^{1}H-NMR spectrum of (Ra)-**2M** in toluene-d8 at the given temperatures (left). Eyring plot of the thermal decay of (Ra)-**2M** **to** (Sa)-**2S**.*

Table S3: *Activation parameters of (Ra)-**2M** to (Sa)-**2S** at 20 °C.*

**ΔG‡$_{293\,K}$ (kJ/mol)**	**ΔG‡$_{293\,K}$ (kcal/mol)**	**Half life (293 K)**
108.5 ± 1.7	25.9 ± 0.4	693 h
**ΔH‡$_{293\,K}$ (kcal/mol)**	**ΔH‡$_{293\,K}$ (kJ/mol)**	
101.8	24.3	
**ΔS‡$_{293\,K}$ (kcal/mol)**	**ΔS‡$_{293\,K}$ (kJ/mol)**	
-22.9	-5.4	

Computational analysis

Computational analysis was carried out using the Gaussian 16, Rev B.01 software package.[7] Motor **2** was optimised considering all of the states involved in the thermal step of a molecular motor, (stable and metastable, with multiple intermediates each connected by a transition state) for both configurations (R_a)-**2**$_S$ and (S_a)-**2**$_S$. All computed structures are provided as .xyz files in a separate Supplementary File. The configurations of the motor were optimised by DFT at the B3LYP/6-31G(d,p) level. All stationary points were confirmed to be such due to the number of imaginary frequencies obtained after the Hessian calculation (0 for minima, 1 for transition states).

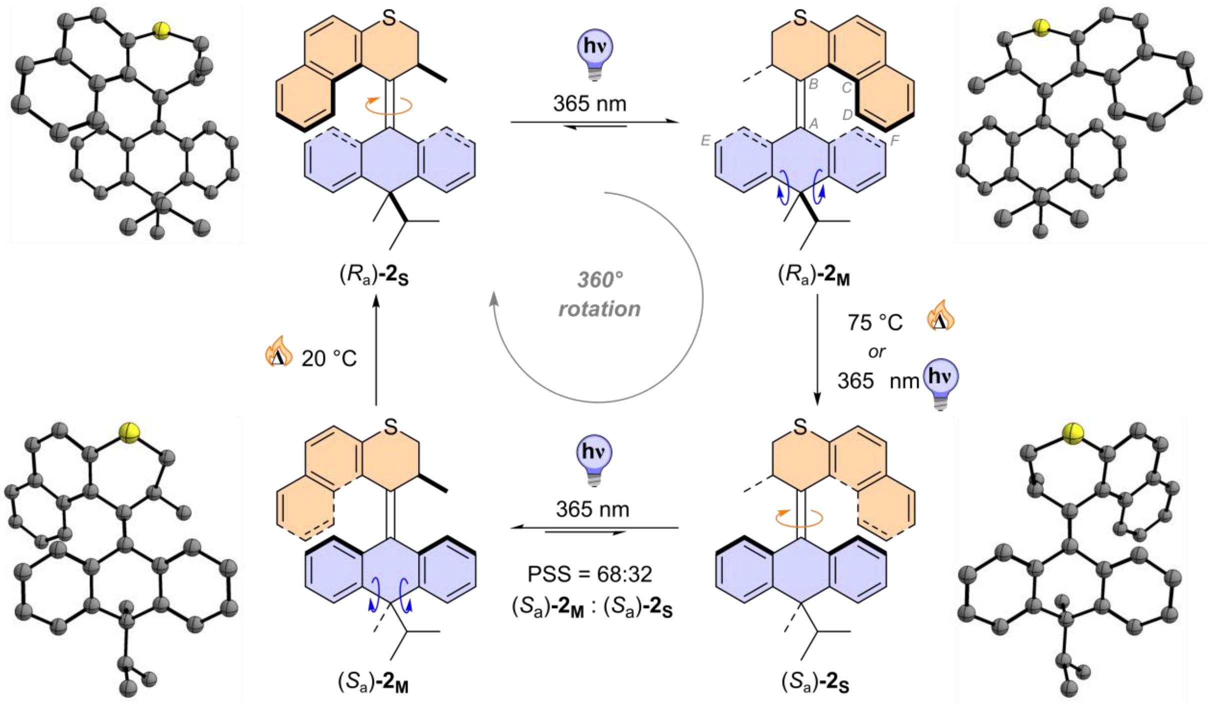

Figure S12: *Rotation cycle of motor **2** with simulated DFT structures depicted.*

Table S4: *Relative energies of motor **2**.*

Motor isomer	Relative energy / kJ mol^{-1}	Dihedral angle / ° (*ABCD*)	Folding angle / ° (*EAF*)
(R_a)-**2**$_S$	0.00	54.00	128.69
(R_a)-**2**$_M$	20.11	56.24	125.77
(S_a)-**2**$_S$	5.80	54.00	128.69
(S_a)-**2**$_M$	46.46	56.24	125.77

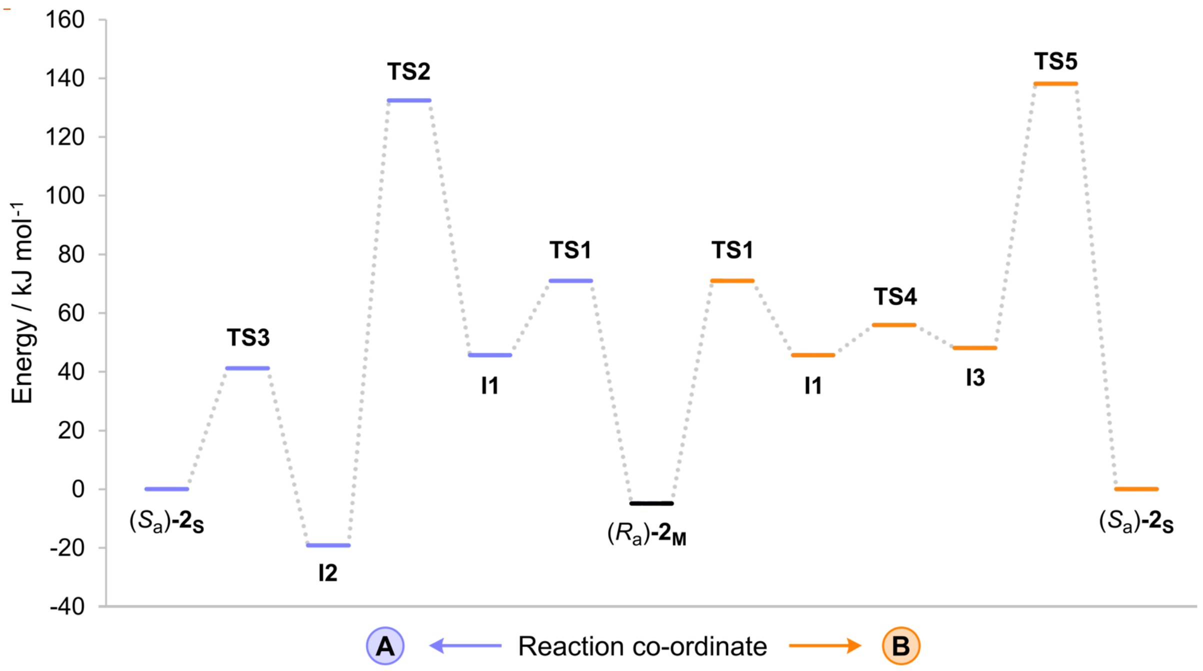

Figure S13: *Energy profile along the reaction co-ordinate for the THI step from (Ra)-**2M** to (Sa)-**2S**.*

Table S5: *Relative energies of the DFT calculated structures during the THI step from (Ra)-**2M** to (Sa)-**2S**.*

Motor isomer	Relative energy / kJ mol^{-1}
(S_a)-**2**$_S$	0.00
**TS3**	41.18
**I2**	-19.19
**TS2**	132.47
**I1**	45.67
**TS1**	71.01
(R_a)-**2**$_M$	-4.88
**TS4**	55.90
**I3**	48.07
**TS5**	138.14

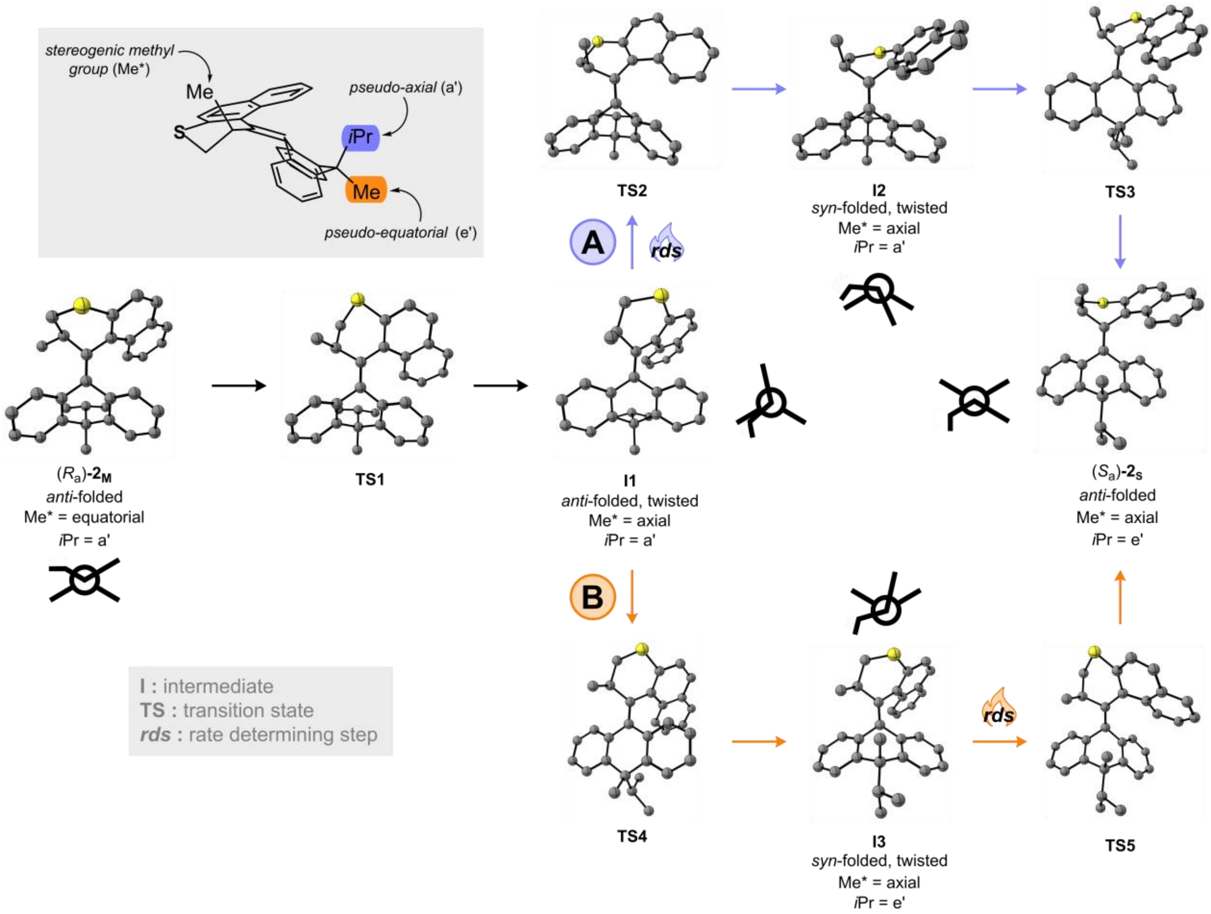

Figure S14: *Thermal conversion from metastable (R_a)-**2$_M$** to stable (S_a)-**2$_S$** via pathways A (top, purple) and B (bottom, orange). With all intermediates a schematic top view of the molecule along the double bond axis is drawn. The rate-determining ring-flip in the upper half is indicated with rds.*

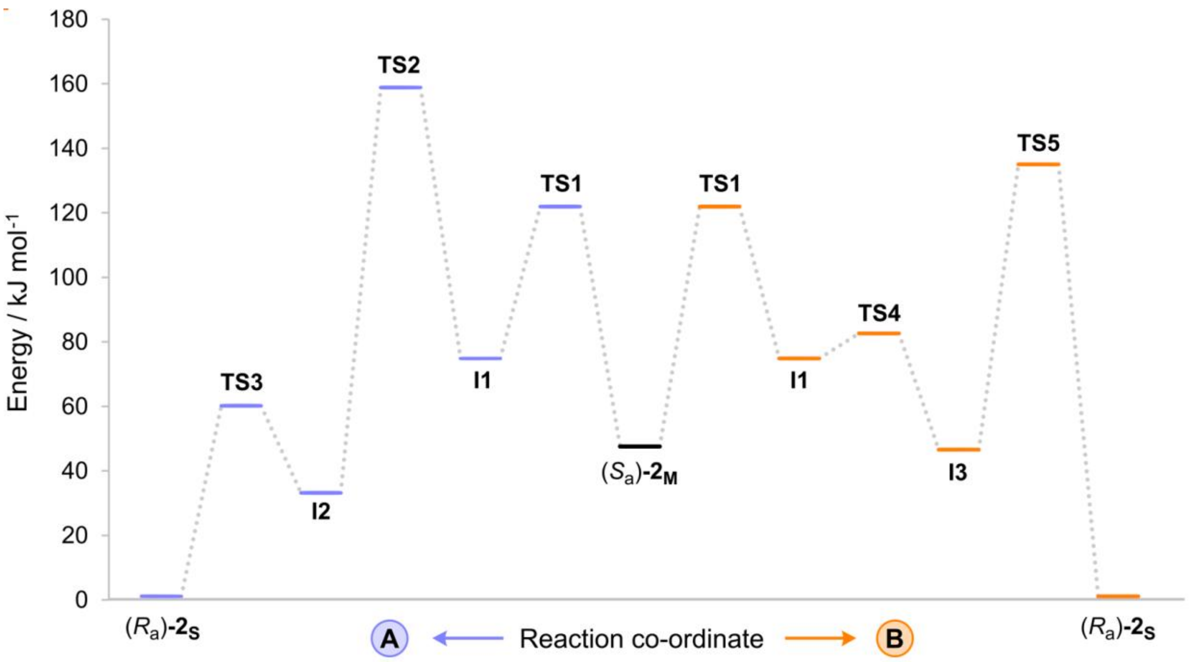

Figure S15: *Energy profile along the reaction co-ordinate for the THI step from (S$_a$)-**2$_M$** to (R$_a$)-**2$_S$**.*

Table S6: *Relative energies of the DFT calculated structures during the THI step from (S$_a$)-**2$_M$** to (R$_a$)-**2$_S$**.*

Motor isomer	Relative energy / kJ mol^{-1}
(*R*$_a$)-**2$_S$**	0.00
**TS3**	59.08
**I2**	32.00
**TS2**	157.73
**I1**	73.76
**TS1**	120.83
(*S*$_a$)-**2$_M$**	46.46
**TS4**	81.43
**I3**	45.44
**TS5**	133.86

Conformational Analysis by NOESY

A detailed analysis in NMR of the two stable isomers was done to confirm the conformations in solution. Interproton distances were derived from DFT calculated 3D models of different possible isomers (*syn*- and *anti*-folded) and matched with the NOESY spectra of (R_a)-$\mathbf{2_S}$ and (S_a)-$\mathbf{2_S}$ in MestreNova. Based on this comparison the conformer with the best fit to the NOESY spectrum was identified. The NOESY data best supports the calculated inter proton distances for the *syn*-folded conformer (Table S7 and Figure S16). All calculated distances match the intensity of the corresponding NOESY signals, as apparent through comparison of the inter proton distances listed in Table S7 and the annotations of the NOESY signals in Figure S16. The *anti*-folded conformer is shown for comparison in Table 80 and Figure S17. The distances derived from the 3D model do not match the recorded spectrum.

Table S7: *Summary of the inter-proton distances determined from DFT calculated structures for the structural elucidation of syn-(R)-**2*****S*** *in NOESY.*

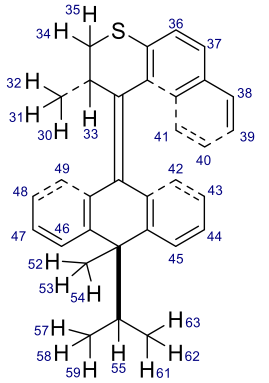

syn-(*S*)-(*P*)-(R_a)-**2**$_\mathbf{S}$

Proton 1	Proton 2	Position (ppm)	Distances ≤3.5 Å
41	42	1.06/8.31	2.5
41	30/31/32	6.88/8.31	3.0
33	30/31/32	1.06/4.25	2.4
33	34	2.96/4.25	2.3
33	35	2.50/4.25	2.6
33	49	4.32/7.73	2.3
46	52/53/54	1.64/7.50	2.1
46	57/58/59	0.73/7.50	3.1
45	52/53/54	1.64/7.50	2.0
45	61/62/63	0.62/7.27	2.9
55	61/62/63	0.62/2.83	2.4
55	57/58/59	0.73/2.83	2.4

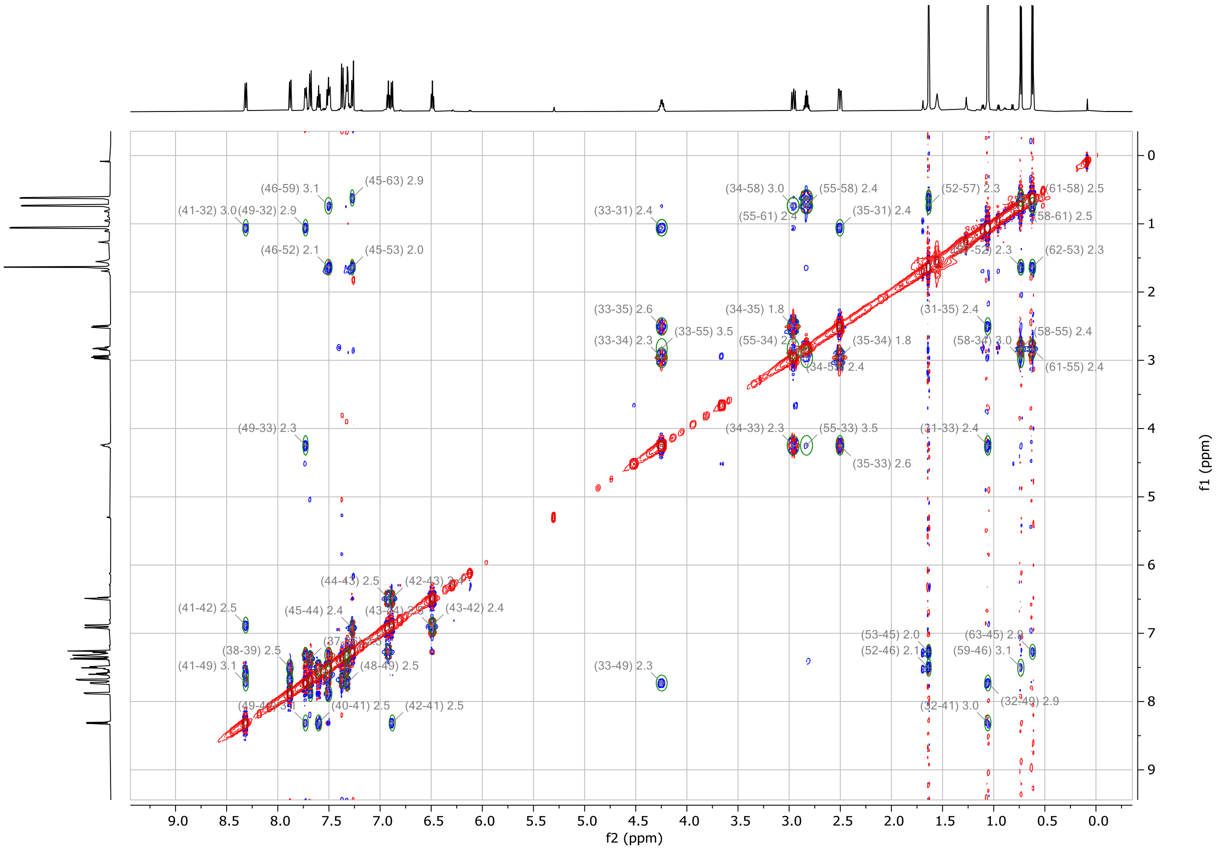

Figure S16: *Experimental NOESY spectrum of (S_a)-**2**$_\mathbf{S}$ overlaid with simulated NOE correlations of syn-folded conformer I2 .*

Table S8: *Summary of the inter-proton distances determined from DFT calculated structures for the structural elucidation of anti-(R)-**2s** in NOESY.*

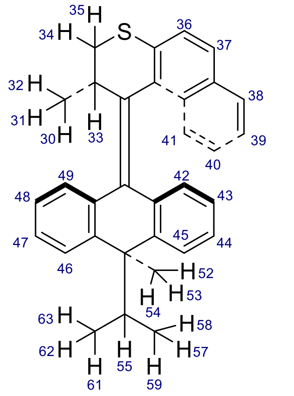

anti-(*S*)-(*P*)-(*S*$_a$)-**2s**

Proton 1	Proton 2	Position (ppm)	Distances ≤3.5 Å
41	42	1.06/8.31	/
41	30/31/32	6.88/8.31	3.0
33	30/31/32	1.06/4.25	2.4
33	34	2.96/4.25	2.3
33	35	2.50/4.25	2.8
33	49	4.32/7.73	2.3
46	52/53/54	1.64/7.50	3.3
46	57/58/59	0.73/7.50	/
45	52/53/54	1.64/7.50	3.4
45	61/62/63	0.62/7.27	2.1
55	61/62/63	0.62/2.83	2.4
55	57/58/59	0.73/2.83	2.4

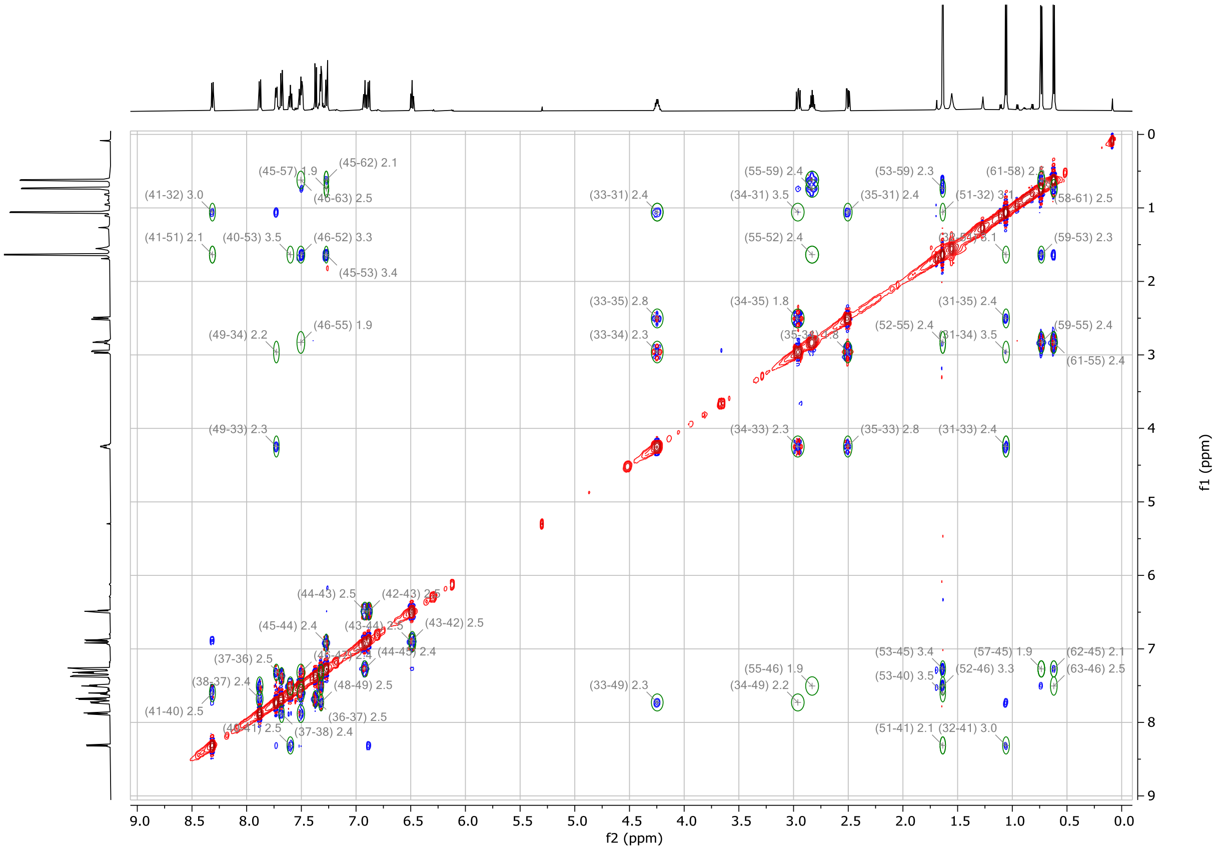

Figure S17: *Experimental NOESY spectrum of (S*$_a$*)-**2s** overlaid with simulated NOE correlations of anti-folded conformer (S*$_a$*)-**2s***

References:

[1] N. Koumura, E. M. Geertsema, M. B. Van Gelder, A. Meetsma, B. L. Feringa, *J. Am. Chem. Soc.* **2002**, *124*, 5037–5051.

[2] R. K. Dhar, D. K. Clawson, F. R. Fronczek, P. W. Rabideau, *J. Org. Chem* **1992**, *57*, 2917–2921.

[3] J. C. M. Kistemaker, S. F. Pizzolato, T. van Leeuwen, T. C. Pijper, B. L. Feringa, *Chem. - A Eur. J.* **2016**, *22*, 13478–13487.

[4] L. Krause, R. Herbst-Irmer, G. M. Sheldrick, D. Stalke, *J. Appl. Crystallogr.* **2015**, *48*, 3–10.

[5] G. M. Sheldrick, *Acta Crystallogr. Sect. A* **2015**, *71*, 3–8.

[6] G. M. Sheldrick, *Acta Crystallogr. Sect. A* **2008**, *64*, 112–122.

[7] M. J. Frisch, G. W. Trucks, H. B. Schlegel, G. E. Scuseria, M. a. Robb, J. R. Cheeseman, G. Scalmani, V. Barone, G. a. Petersson, H. Nakatsuji, et al., **2016**, Gaussian 16, Revision C.01, Gaussian, Inc., Wallin.

NMR spectra

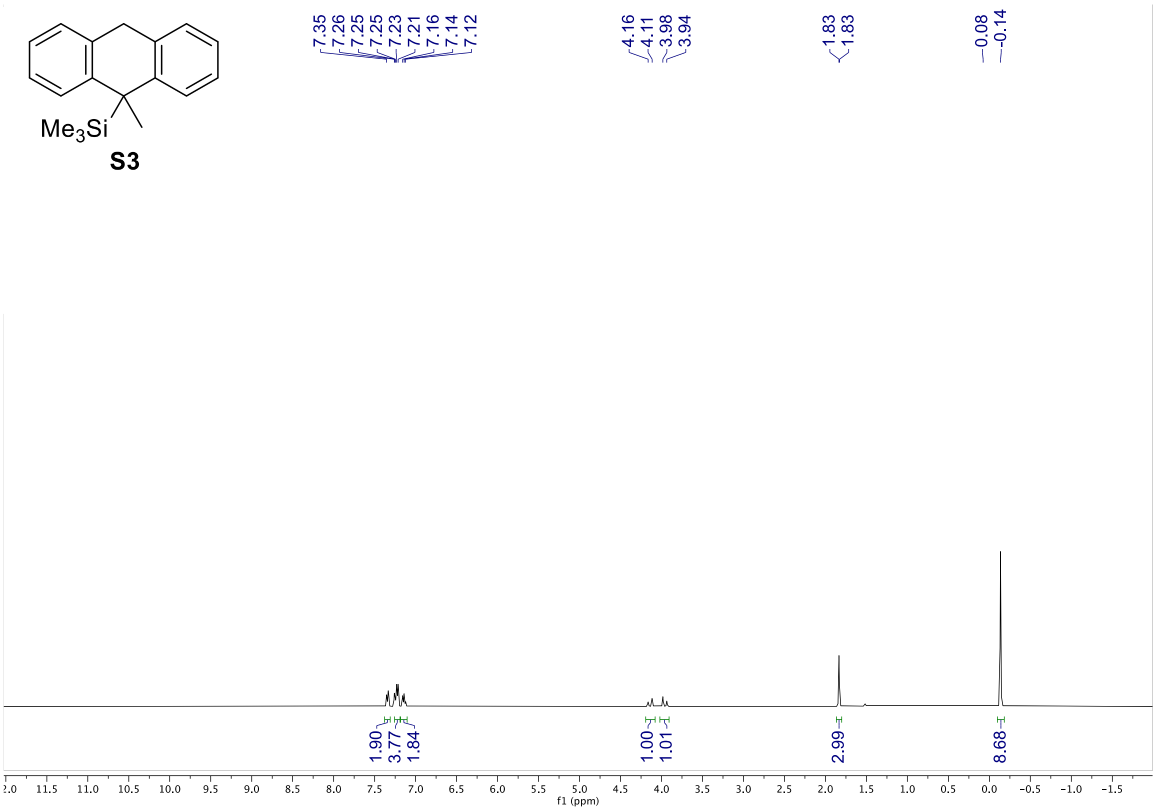

^{1}H NMR (400 MHz) of Trimethyl(9-methyl-9,10-dihydroanthracen-9-yl)silane (**S3**) in $CDCl_3$

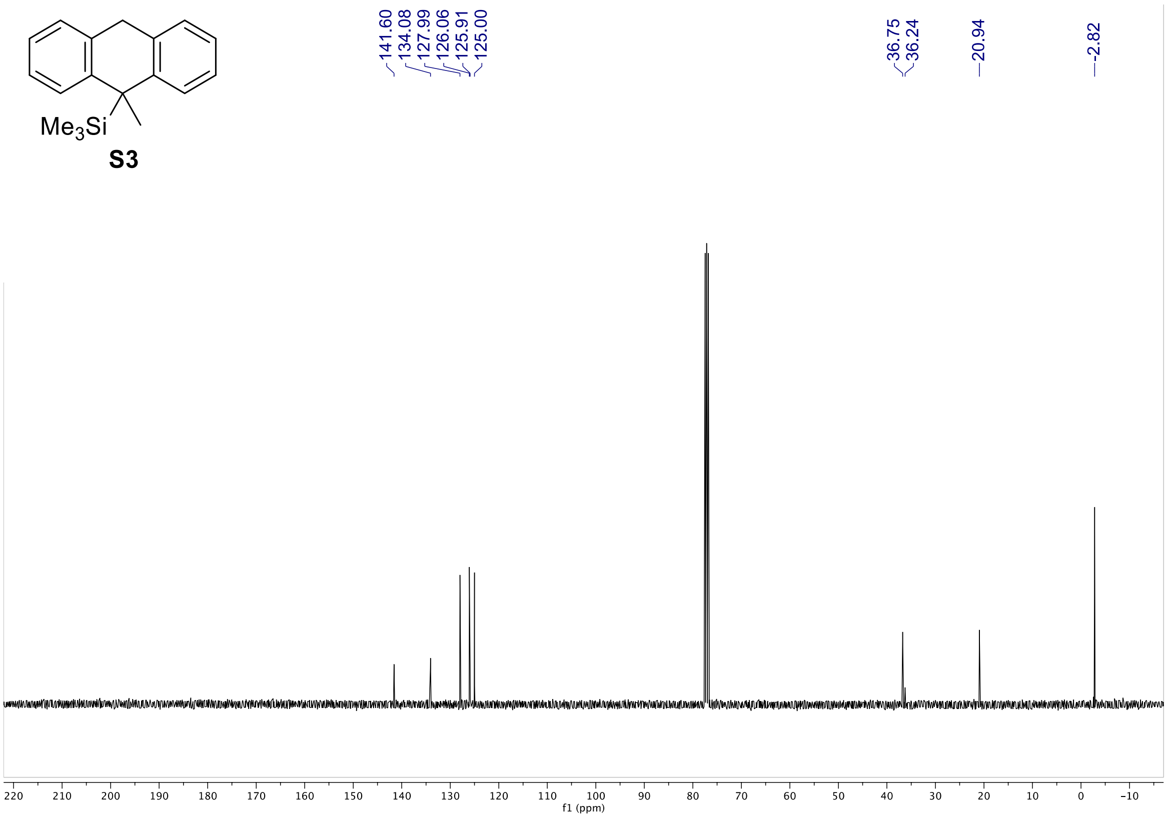

$^{13}C\{^1H\}$ NMR (101 MHz) of Trimethyl(9-methyl-9,10-dihydroanthracen-9-yl)silane (**S3**) in $CDCl_3$

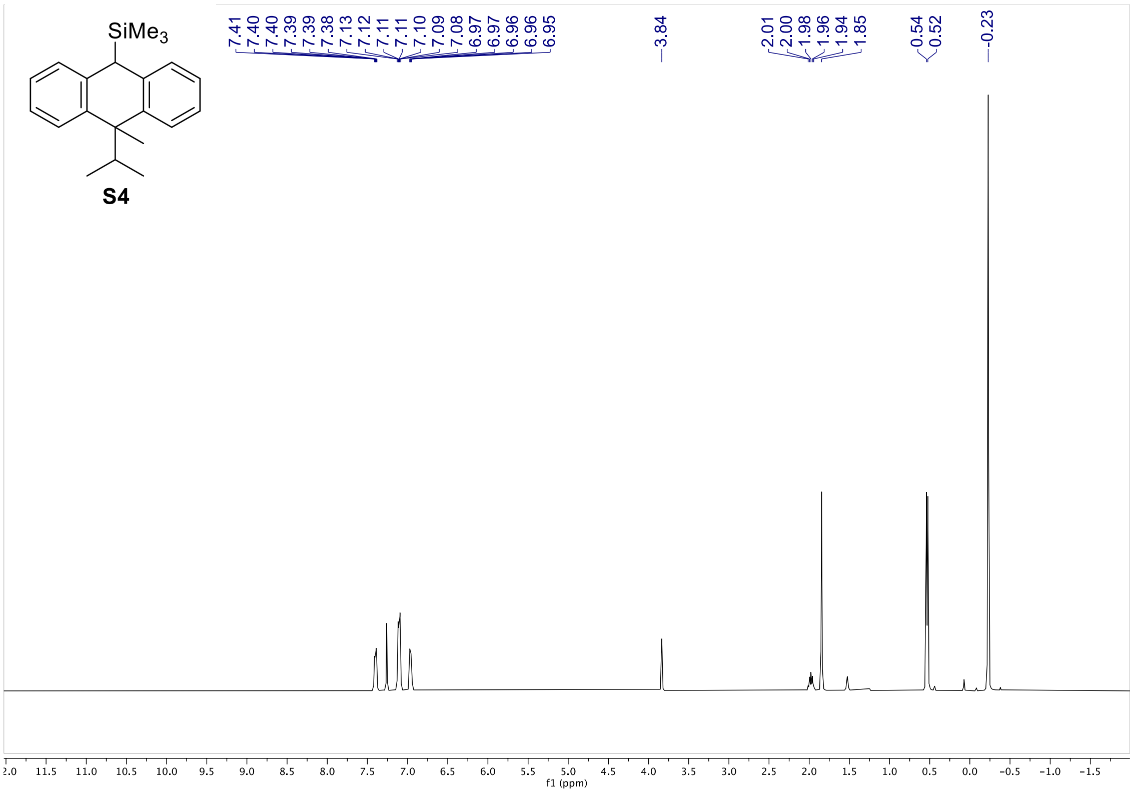

^{1}H NMR (400 MHz) of (10-Isopropyl-10-methyl-9,10-dihydroanthracen-9-yl)trimethylsilane (**S4**) in $CDCl_3$

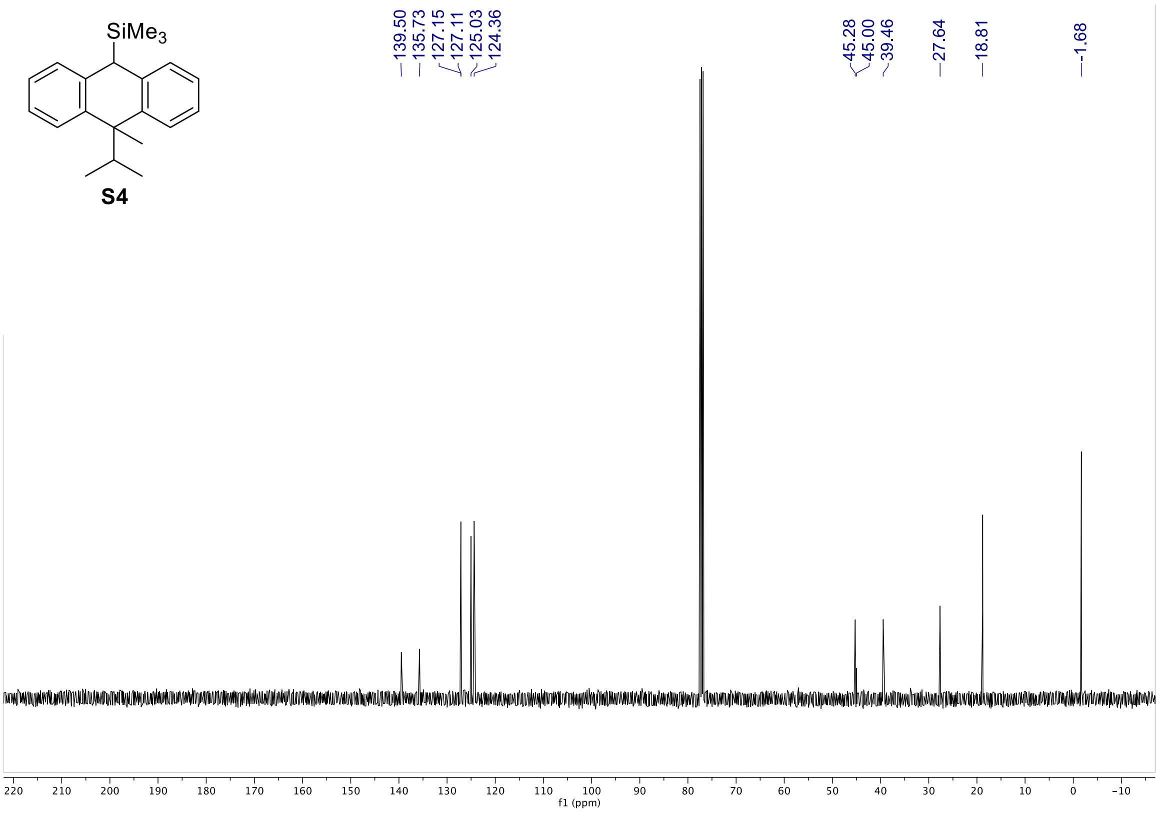

$^{13}C\{^1H\}$ NMR (101 MHz) of (10-Isopropyl-10-methyl-9,10-dihydroanthracen-9-yl)trimethylsilane (**S4**) in $CDCl_3$

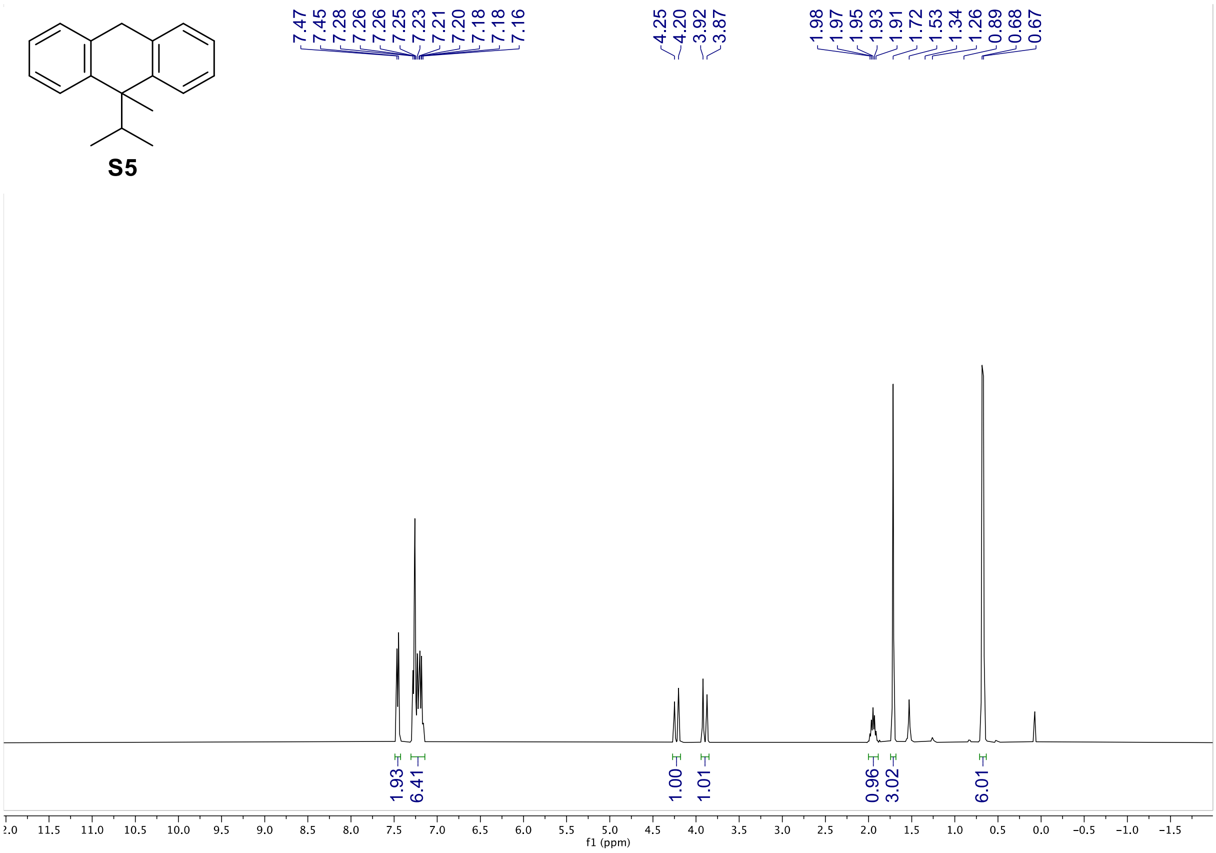

^{1}H NMR (400 MHz) of 9-Isopropyl-9-methyl-9,10-dihydroanthracene (**S5**) in $CDCl_3$

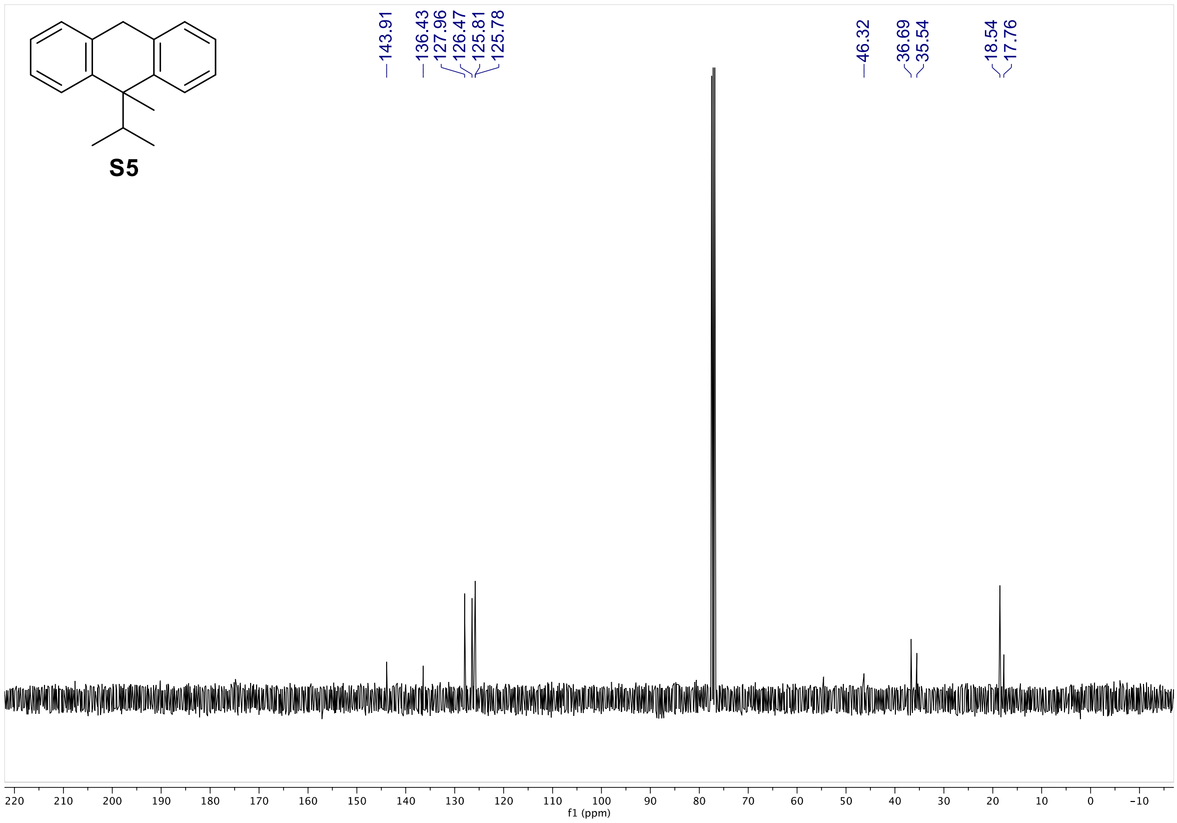

$^{13}C\{^{1}H\}$ NMR (101 MHz) of 9-Isopropyl-9-methyl-9,10-dihydroanthracene (**S5**) in $CDCl_3$

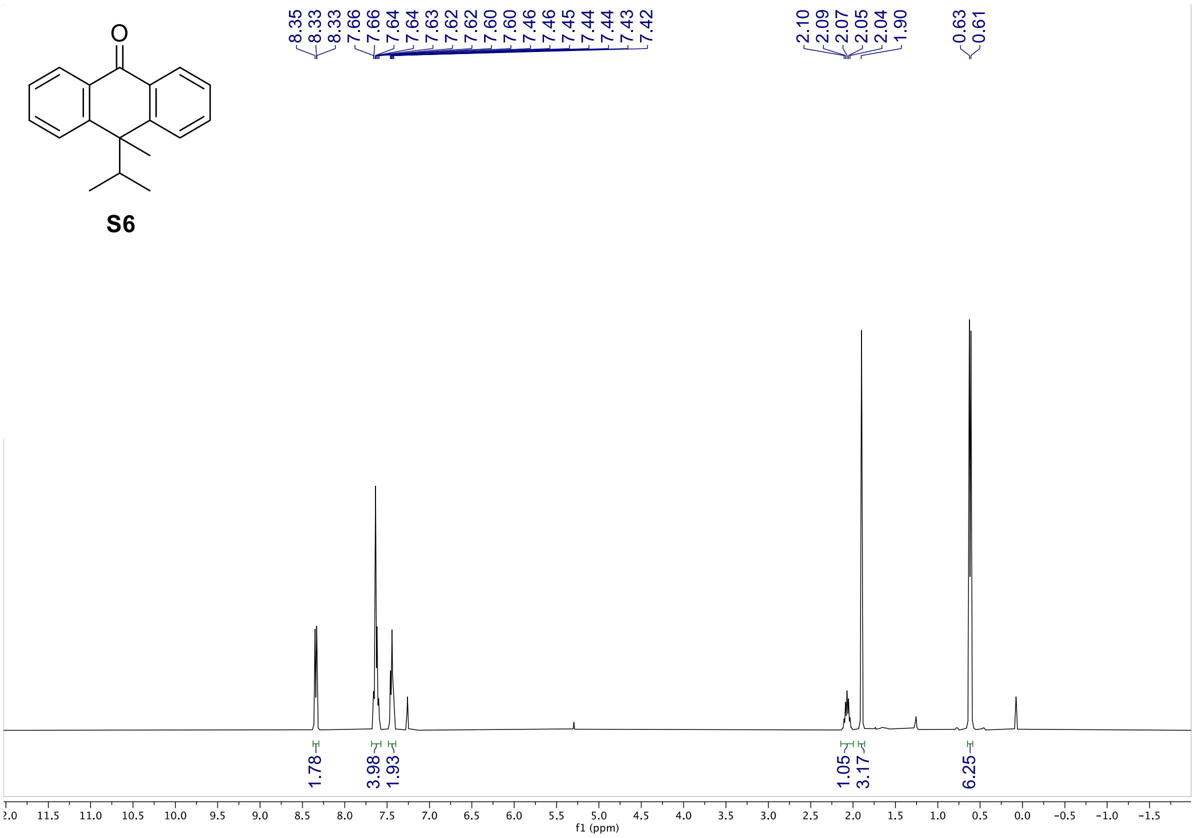

^{1}H NMR (400 MHz) of 10-Isopropyl-10-methylanthracen-9(10*H*)-one (**S6**) in $CDCl_3$

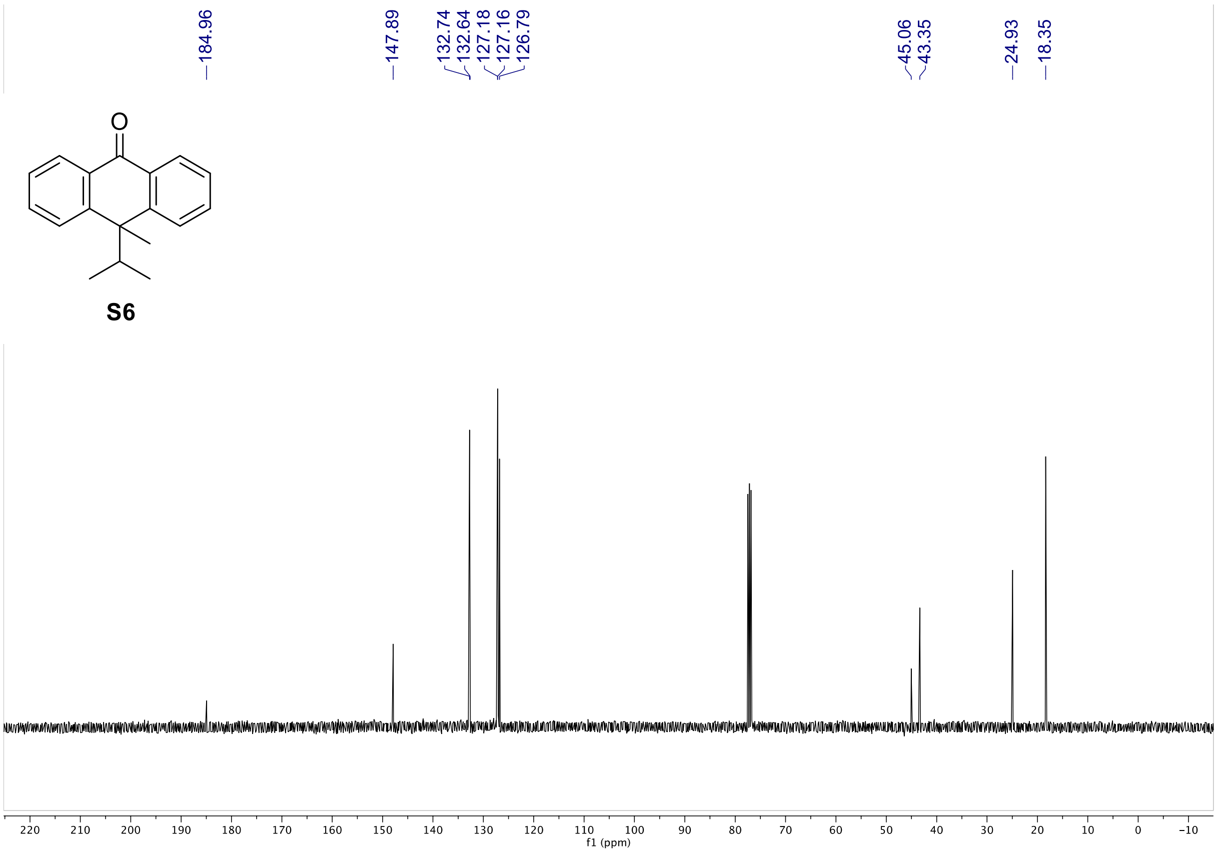

$^{13}C\{^{1}H\}$ NMR (101 MHz) of 10-Isopropyl-10-methylanthracen-9(10*H*)-one (**S6**) in $CDCl_3$

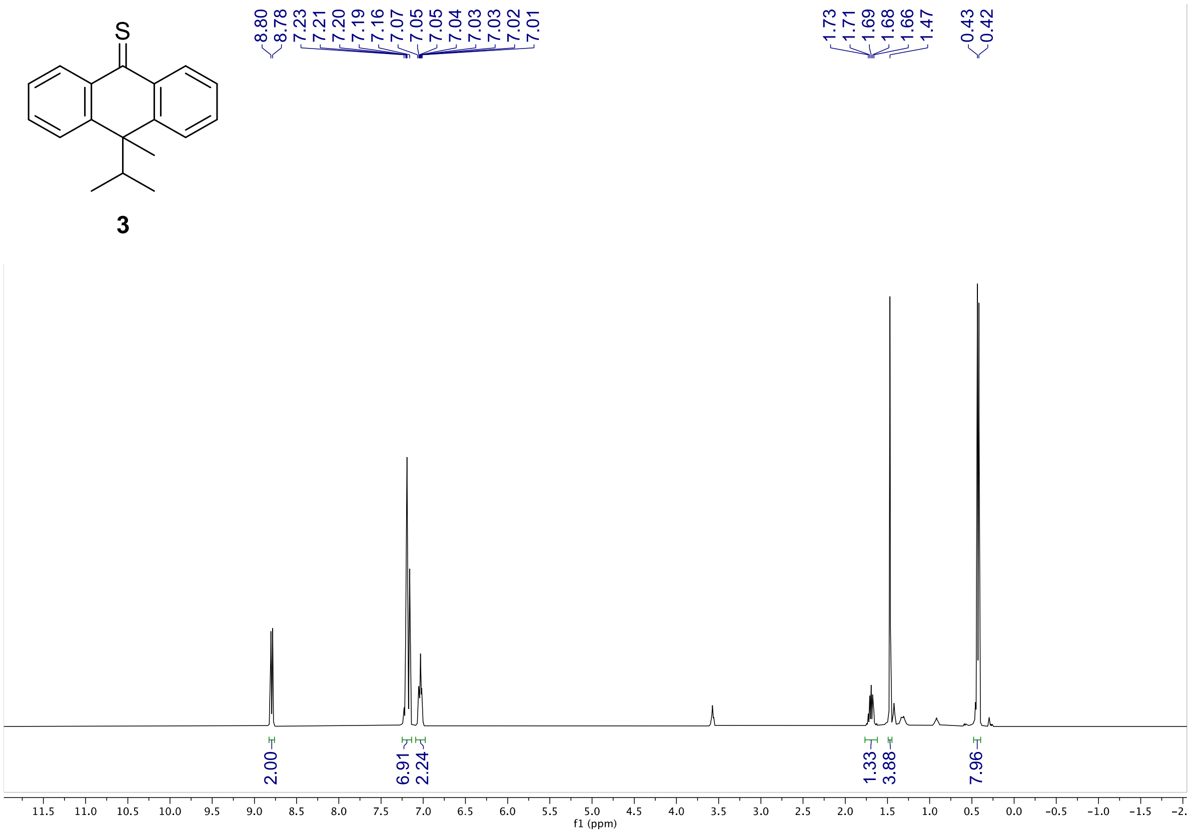

^{1}H NMR (400 MHz) of 10-Methyl-10-isopropyl-anthracen-9-thione (**3**) in C_6D_6

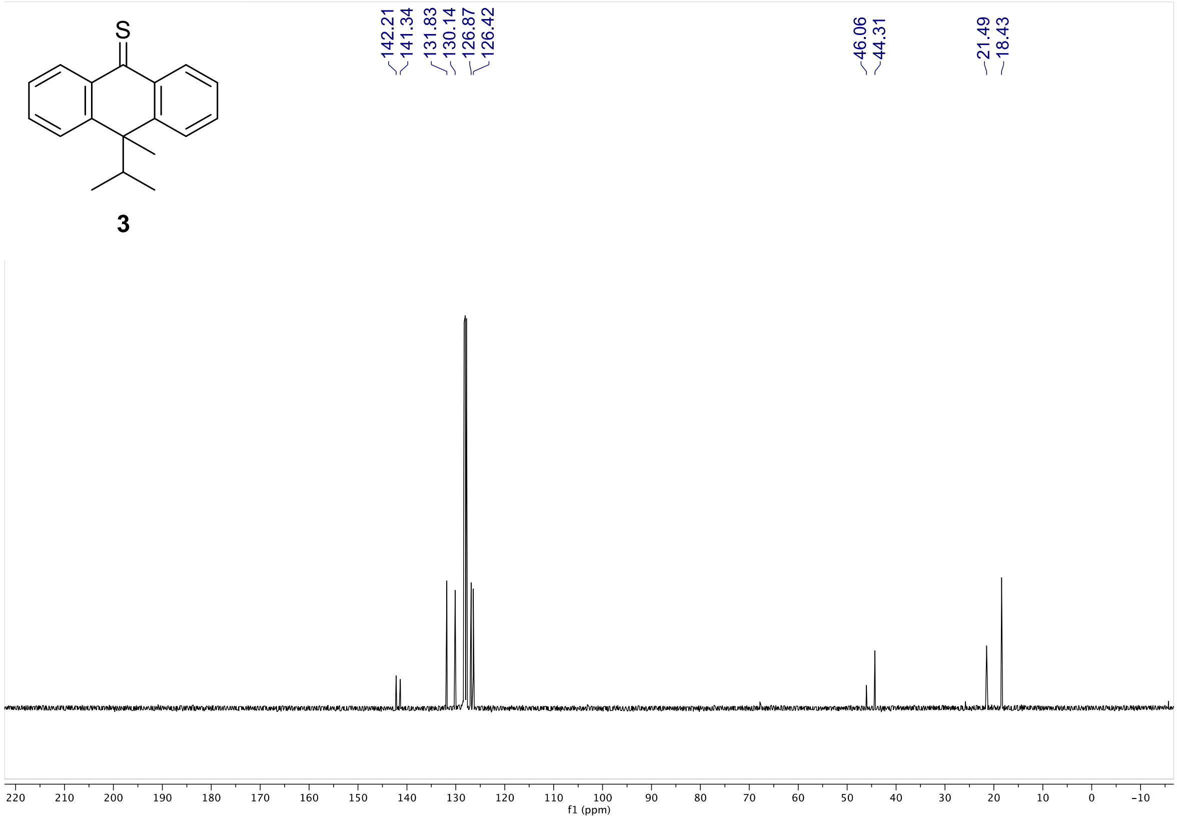

^{13}C{^{1}H} NMR (101 MHz) of 10-Methyl-10-isopropyl-anthracen-9-thione (**3**) in C_6D_6

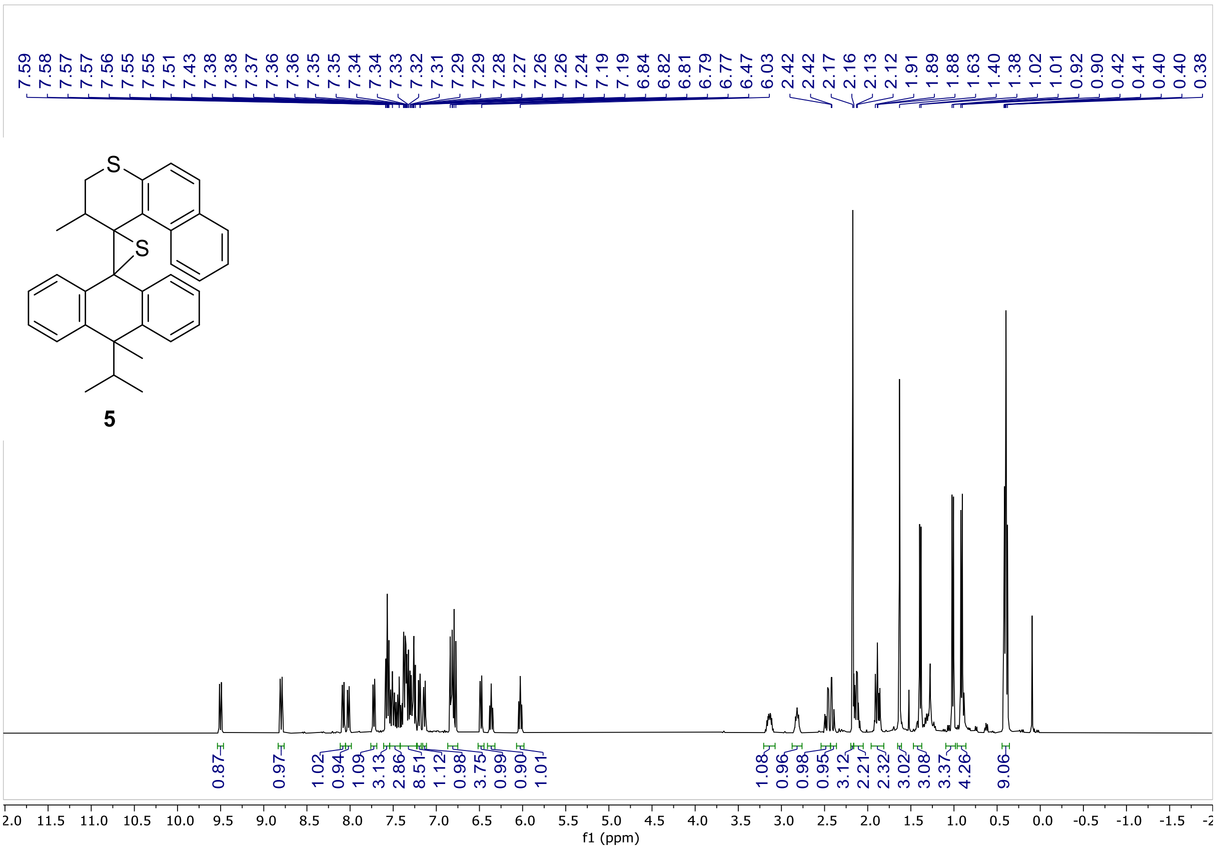

^{1}H NMR (400 MHz) of Isomeric mixture of episulfide **5** in $CDCl_3$

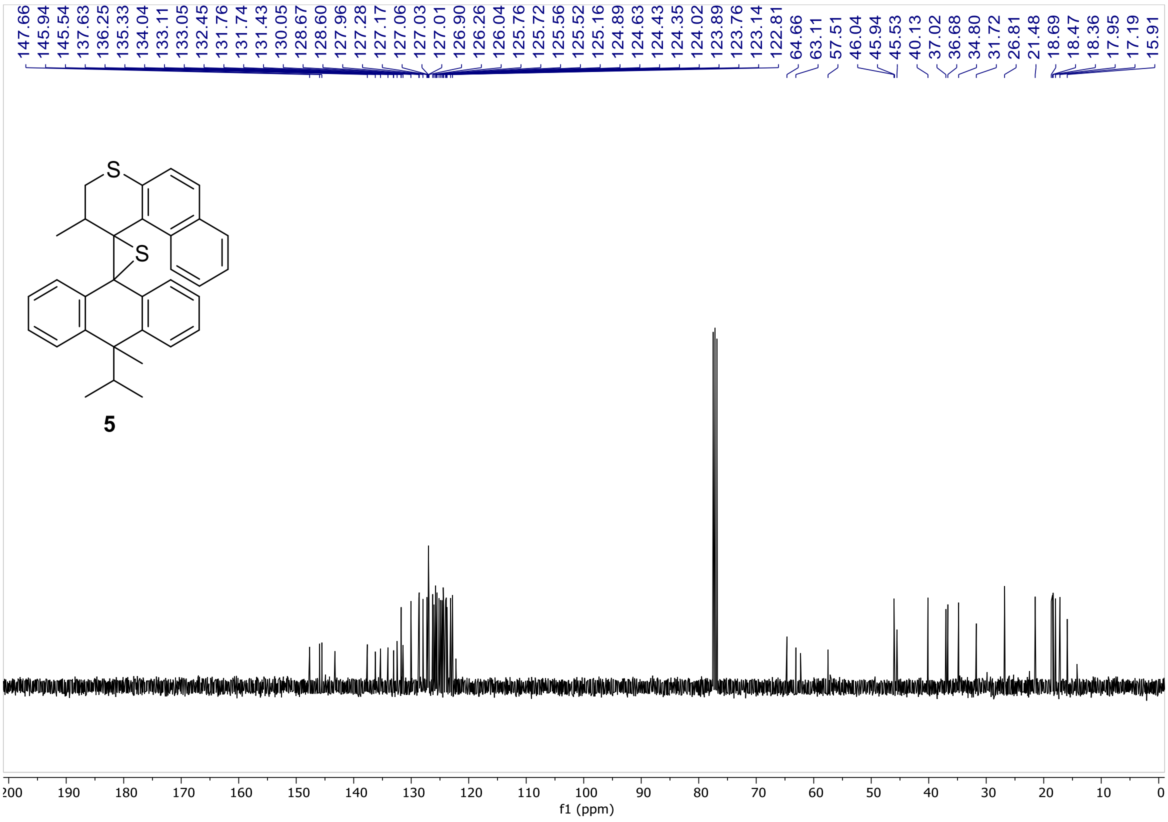

$^{13}C\{^{1}H\}$ NMR (101 MHz) of Isomeric mixture of episulfide **5** in $CDCl_3$

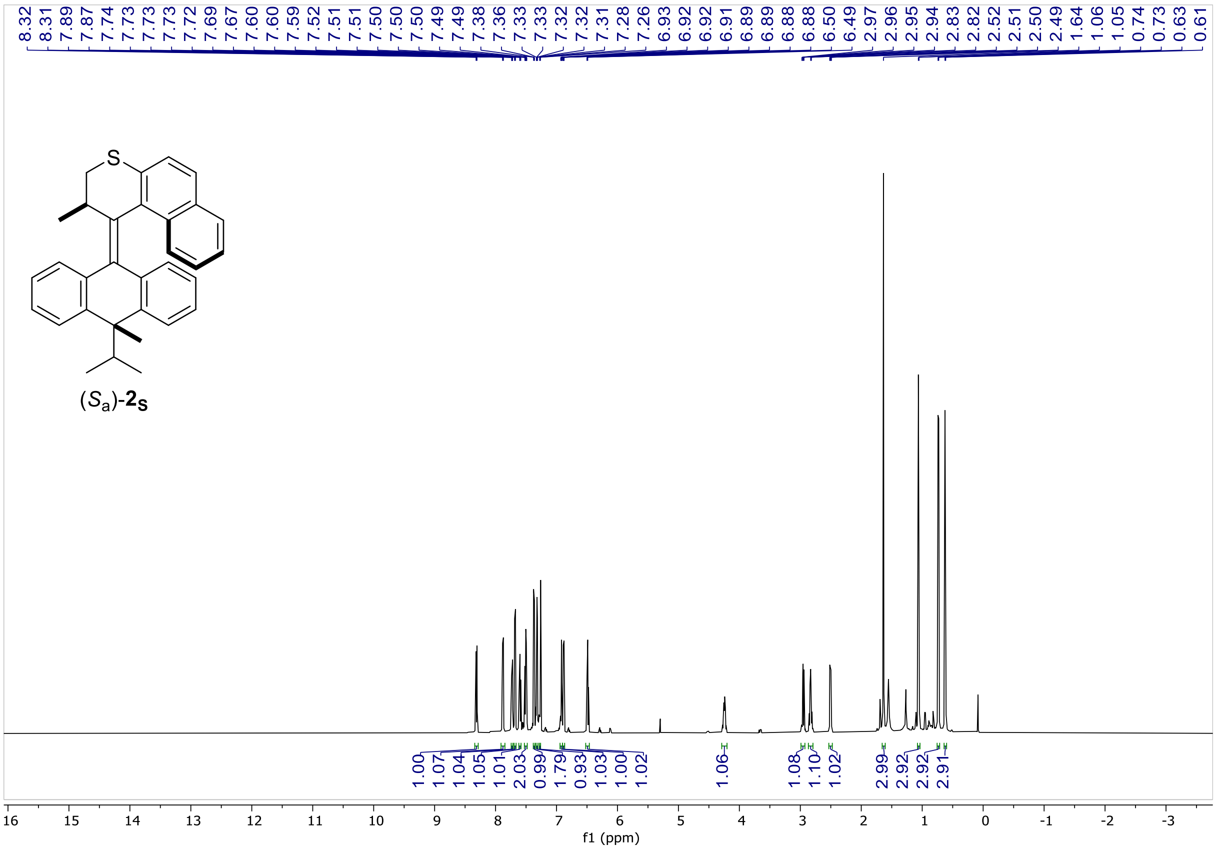

^{1}H NMR (600 MHz) of Isomer (S_a)-$\mathbf{2_S}$ of molecular motor in $CDCl_3$

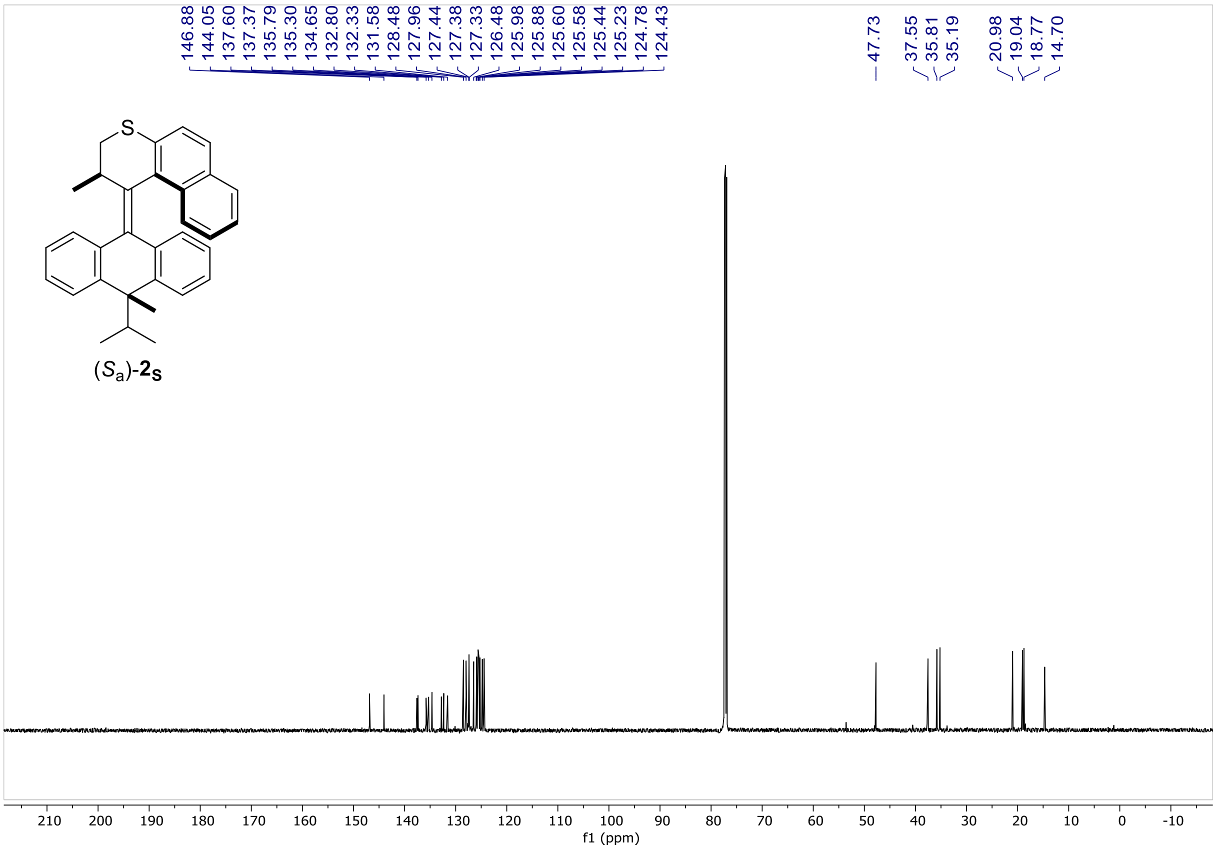

^{13}C{^{1}H} NMR (151 MHz) of Isomer (S_a)-$\mathbf{2_S}$ of molecular motor in $CDCl_3$

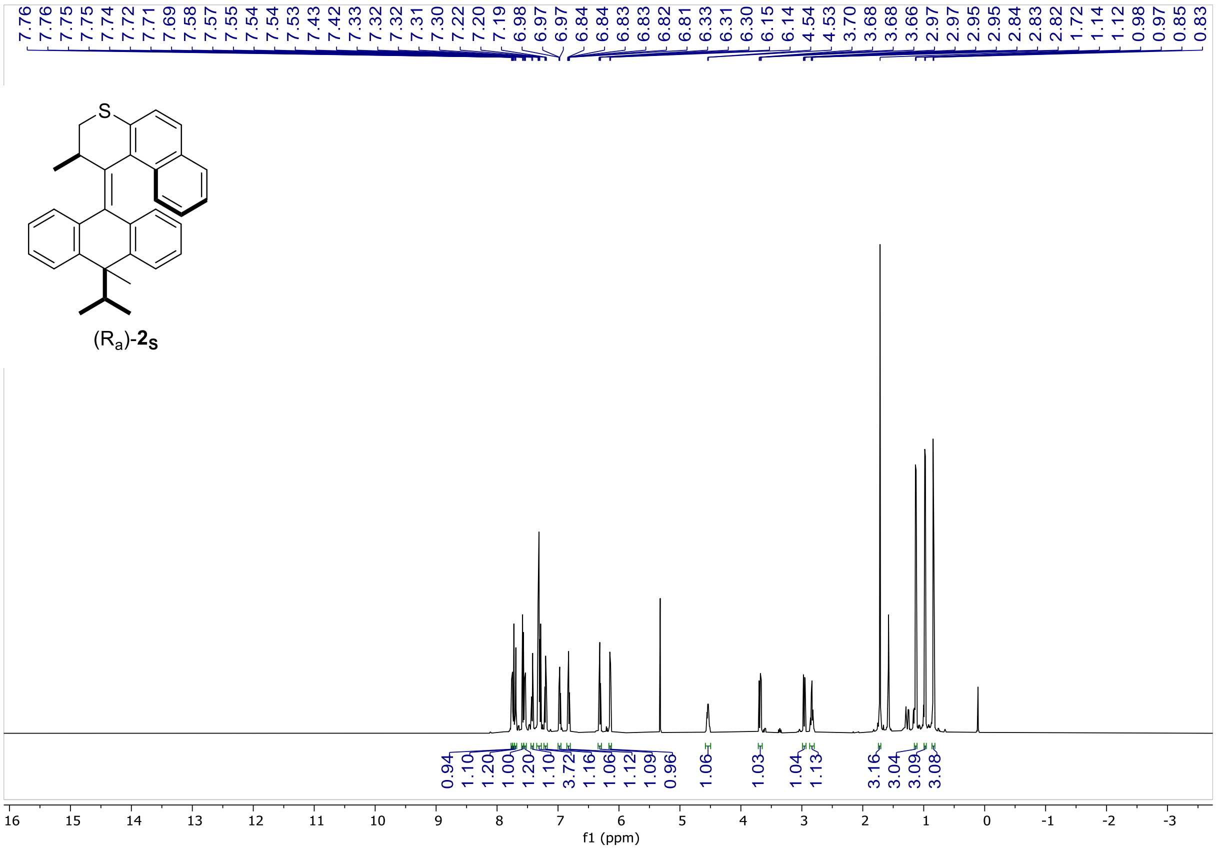

^{1}H NMR (600 MHz) of Isomer (*R*$_a$)-**2$_S$** of molecular motor in $CDCl_3$

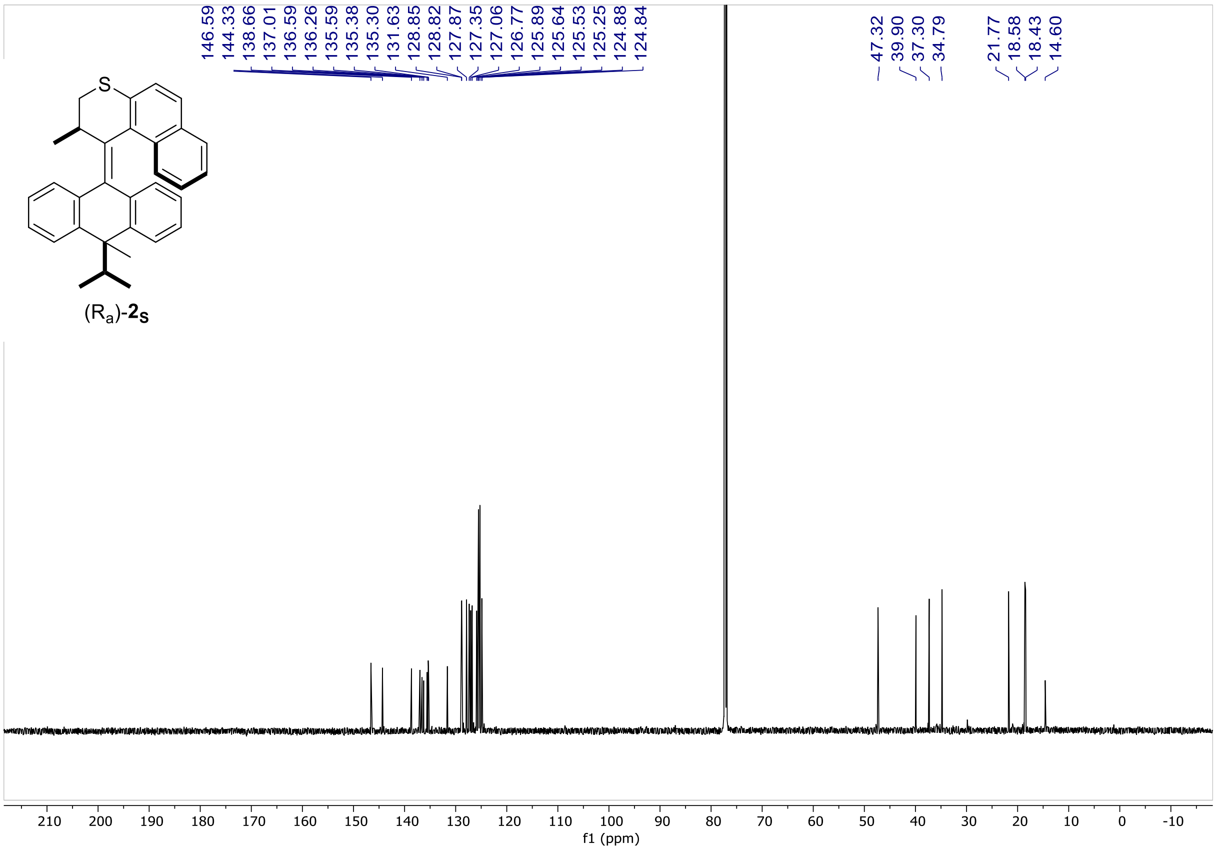

^{13}C{^{1}H} NMR (151 MHz) of Isomer (*R*$_a$)-**2$_S$** of molecular motor in $CDCl_3$

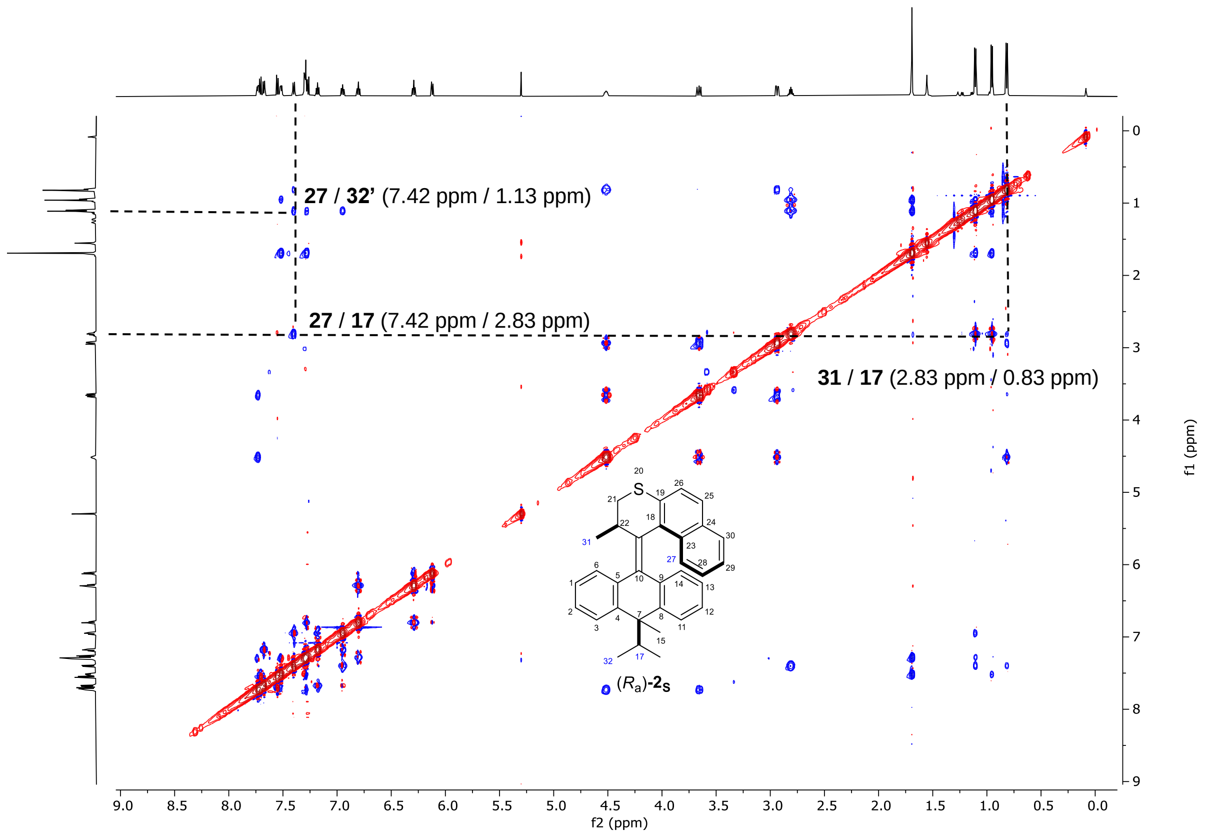

NOESY-NMR of (R_a)-**2**$_S$ in $CDCl_3$ with indication of relevant cross peaks for the assignment of isomers

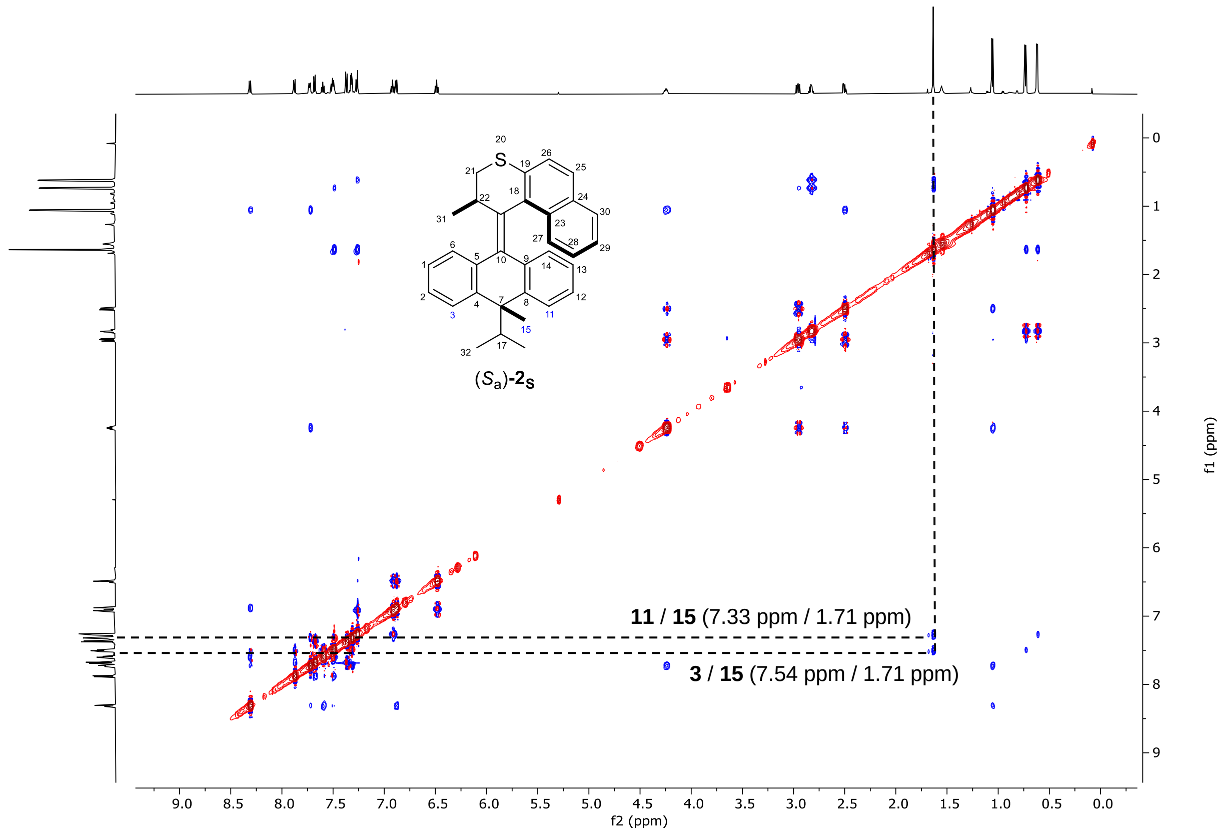

NOESY-NMR of (R_a)-**2**$_S$ in $CDCl_3$ with indication of relevant cross peaks for the assignment of isomers
